# Intramolecular hydrogen-bonding in a cobalt aqua complex and electrochemical water oxidation activity†
†Electronic supplementary information (ESI) available: General experimental details, synthetic methods, characterization, electrochemical data, gas chromatography, cif files. CCDC 1586338–1586341. For ESI and crystallographic data in CIF or other electronic format see DOI: 10.1039/c7sc04960a


**DOI:** 10.1039/c7sc04960a

**Published:** 2018-02-06

**Authors:** Juliet F. Khosrowabadi Kotyk, Caitlin M. Hanna, Rebecca L. Combs, Joseph W. Ziller, Jenny Y. Yang

**Affiliations:** a Department of Chemistry , University of California , Irvine , USA . Email: j.yang@uci.edu

## Abstract

Water oxidation is catalysed in Nature by a redox cofactor embedded in a hydrogen-bonded network designed to orchestrate proton transfer throughout the challenging 4 electron reaction. In order to mimic aspects of this microenvironment, [CoL^DMA^(CH_3_CN)_2_][BF_4_]_2_ (**2**) was synthesized, where L^DMA^ is a dipyridyldiamine ligand with two dimethylamine bases in the secondary coordination sphere. Structural characterization of the corresponding aqua complexes establish hydrogen bonding between the bound water and pendant base(s). Cyclic voltammetry of [CoL^DMA^(CH_3_CN)_2_][BF_4_]_2_ (**2**) reveals enhanced oxidative current upon titration with water and controlled potential electrolysis confirms evolution of O_2_. The related complex [CoL^H^(CH_3_CN)_2_][BF_4_]_2_ (**1**), which has the same primary coordination environment as **2** but lacks pendant bases, is inactive. The structural and electrochemical studies illustrate the role positioned proton relays can play in promoting redox reactivity.

## Abstract 2

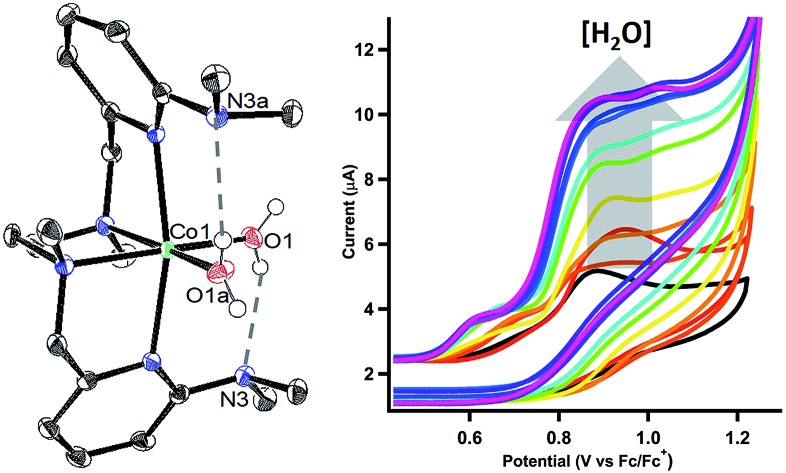
Water oxidation is catalysed in Nature by a redox cofactor embedded in a hydrogen-bonded network designed to orchestrate proton transfer throughout the challenging 4 electron reaction.

## Introduction

In Nature, the Oxygen Evolving Complex (OEC) in photosystem II catalyses the oxidation of water to liberate proton and electron equivalents for carbon dioxide reduction. The challenging 4-electron, 4-proton reaction is choreographed using a precisely tuned network of hydrogen-bonding interactions.^1^–^5^ Efforts to design synthetic catalysts that mimic aspects of this microenvironment have primarily utilized distal interactions to orient water for O–O bond formation. These interactions have either been incorporated into the ligand,^6^–^11^ or are assembled through the use of appropriate buffers.^12^

Proximal interactions are also important for managing proton inventory at the reaction site. *Sequential* removal of protons with electrons enables successive oxidation events to occur in a smaller potential window (redox-levelling). The magnitude of this effect has been quantified in synthetic models.^13^,^14^ *Concerted* proton and electron transfer provides an even lower energetic route for water oxidation and can be achieved through proton acceptors of correct positioning and p*K*_b_.^15^–^17^ This mechanism is believed to be important to the OEC, which performs the rapid four-electron oxidation of water within a narrow 0.3 V range.^18^–^24^ Concerted proton transfer is also cited in the synthetic catalyst [Ru^II^(damp)(bpy)(H_2_O)]^2+^, which orients external water molecules to couple proton removal with O–O bond formation.^25^,^26^

Constructing a synthetic scaffold to facilitate concerted proton transfer presents challenges as the base must be positioned and matched to both the substrate and reaction conditions. For example, appropriately positioned bases were previously installed in a series of iron complexes. Their activity for water oxidation was investigated using the chemical oxidant Ce(iv) at very low pH. Under these conditions the bases were likely already protonated, and negligible changes in activity were observed.^27^ Likewise, Ru complexes modified with naphthyridine-functionalized ligands display no improvement in activity;^28^–^30^ spectrophotometric titration established that the intended base is a poor proton acceptor as it is unprotonated at pH values as low as zero.^30^ Two copper complexes,^31^,^32^ Cp*Ir(pyalc)Cl,^33^ and most recently a Ru complex^34^ represent the few synthetic examples where proximal proton acceptors contribute to enhance water oxidation activity.

**Chart 1:**
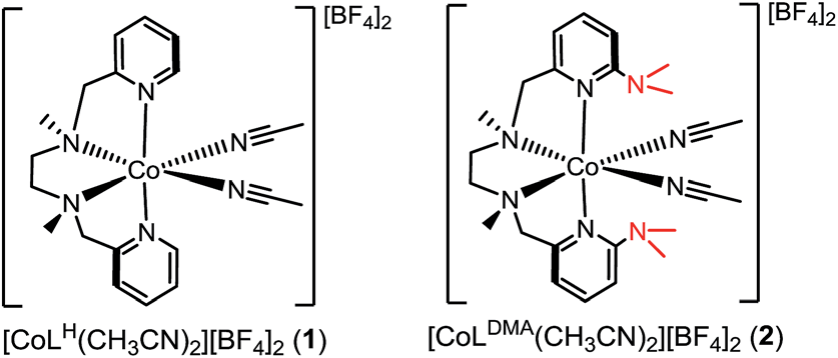


Our interest in concerted proton transfer for small molecule redox catalysis led us to synthesize a series of *N*,*N*′-dimethyl-*N*,*N*′-bis(2-pyridylmethyl)ethane-1,2-diamine (BPMEN) ligands with pendant bases in the secondary coordination sphere.^27^,^35^ In this study, we focused our efforts on [CoL^DMA^(CH_3_CN)_2_]^2+^ (**2**, Chart 1), where L^DMA^ is a BPMEN ligand modified with two dimethylamine pendant bases. In [CoL^DMA^(CH_3_CN)_2_]^2+^ (**2**), the location and p*K*_b_ of the pendant bases are matched for concerted proton transfer upon oxidation of aqua ligands in the primary coordination sphere (*vide infra*). Our choice was supported by structural studies establishing intramolecular hydrogen-bonding interactions in Co(ii) L^DMA^ aqua complexes. Additionally, electrochemical studies demonstrate **2** is competent for water oxidation while [CoL^H^(CH_3_CN)_2_]^2+^ (**1**, Chart 1), which lacks pendant bases, is inactive, supporting the role proximal proton acceptors can play in enabling redox reactivity.

## Results and discussion

### Synthetic procedures

The divalent cobalt complexes [CoL^H^(CH_3_CN)_2_][BF_4_]_2_ (**1**) and [CoL^DMA^(CH_3_CN)_2_][BF_4_]_2_ (**2**) shown in Chart 1 were synthesized by adding the respective ligands to an acetonitrile solution of [Co(CH_3_CN)_6_][BF_4_]_2_,^36^ and were isolated as orange solids in 78–89% yield. The formulations were confirmed by ESI-MS and purity by elemental analysis (see ESI†). Complexes **1** and **2** were also characterized by electron absorption (Fig. S1†) and electron paramagnetic resonance spectroscopy (Fig. S2†).

### Crystallography

**Fig. 1:**
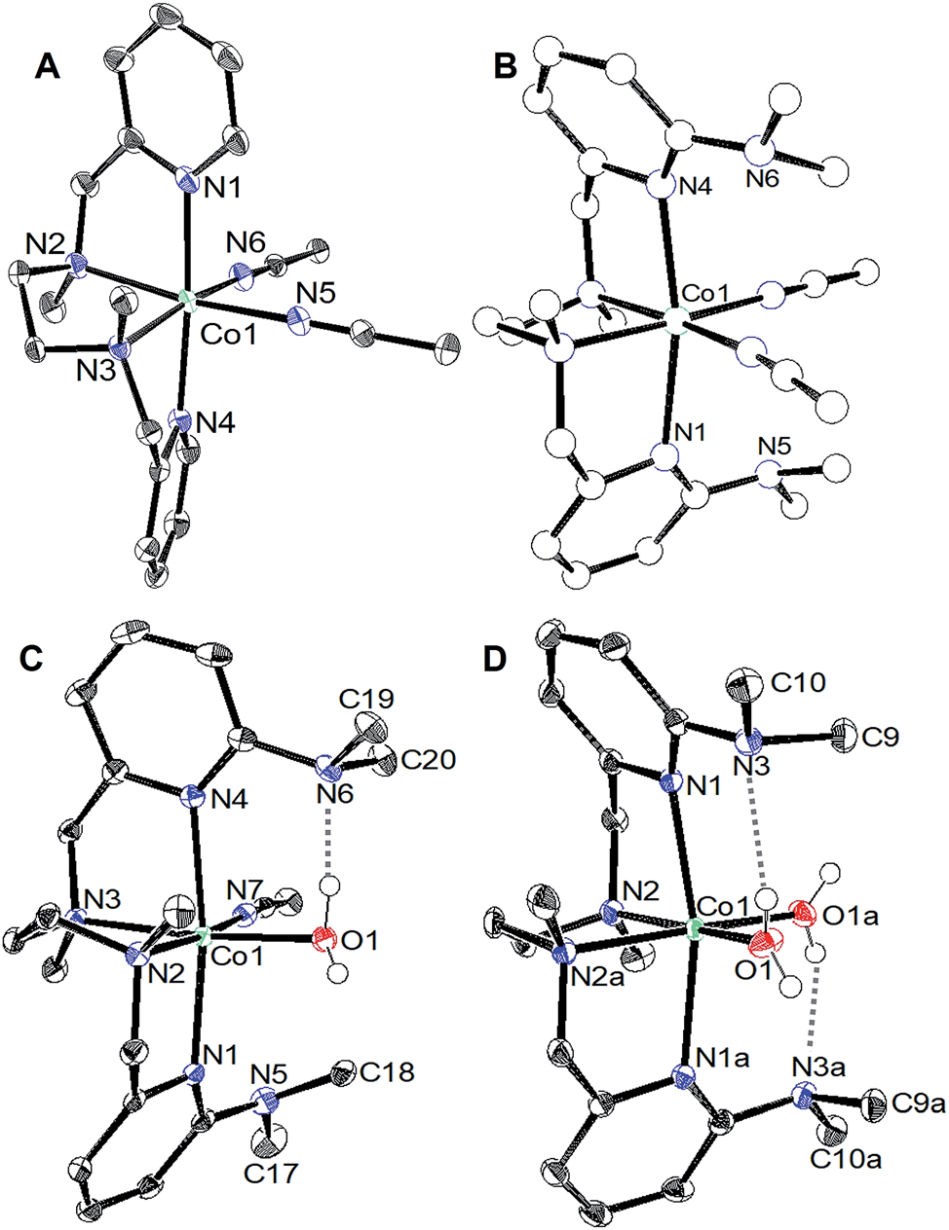
ORTEP of the cobalt complexes (a) [CoL^H^(CH_3_CN)_2_][BF_4_]_2_ (**1**), (c) [CoL^DMA^(CH_3_CN)(H_2_O)][BF_4_]_2_, and (d) [CoL^DMA^(H_2_O)_2_][BF_4_]_2_. Thermal ellipsoids are drawn at the 50% probability level. Gray dashed lines indicate hydrogen-bonding interactions. (b) The quality of the structure for [CoL^DMA^(CH_3_CN)_2_][BF_4_]_2_ (**2**) was poor; a ball and stick diagram is used to depict connectivity. The BF_4_^–^ counter anions, solvent molecules and hydrogen atoms other than those on the aqua ligands have been removed for clarity.

Crystals suitable for single crystal X-ray diffraction were grown by diethyl ether diffusion into acetonitrile. The solid-state structures for **1** and **2** are shown in Fig. 1a and b, respectively. The quality of the diffraction data for **2** was poor, and is only depicted to establish connectivity. As expected, the dimethylamine functional groups in [CoL^DMA^(CH_3_CN)_2_][BF_4_]_2_ (**2**) are poised over the labile coordination sites occupied by solvent.

Diethyl ether diffusion into a mixed H_2_O/CH_3_CN solution of [CoL^DMA^(CH_3_CN)_2_][BF_4_]_2_ (**2**) resulted in crystallization of the mono-aqua complex [CoL^DMA^(CH_3_CN)(H_2_O)][BF_4_]_2_. The corresponding bis-aqua complex [CoL^DMA^(H_2_O)_2_][BF_4_]_2_ was isolated by diffusing diethyl ether into a 5 mL solution of [CoL^DMA^(CH_3_CN)_2_][BF_4_]_2_ (**2**) in the less coordinating solvent, CH_2_Cl_2_ with 1.1 μL H_2_O (2 equiv.). The solid state structures of [CoL^DMA^(CH_3_CN)(H_2_O)][BF_4_]_2_ and [CoL^DMA^(H_2_O)_2_][BF_4_]_2_ are shown in Fig. 1c and d, respectively, and selected bond angles are listed in Table 1. The hydrogen atoms on the aqua ligand(s) were located in the difference map. Although the products were isolated as crystals suitable for X-ray analysis, the complexes turned from pink to blue under reduced pressure, suggesting desolvation^37^ and precluded isolation of the aqua complexes for further characterization.

**Table 1:** Selected bond angles (°) and distances (Å) for [CoL^H^(CH_3_CN)_2_][BF_4_]_2_ (**1**), [CoL^DMA^(CH_3_CN)(H_2_O)][BF_4_]_2_, and [CoL^DMA^(H_2_O)_2_][BF_4_]_2_

[CoL^DMA^(CH_3_CN)(H_2_O)][BF_4_]_2_		[CoL^DMA^(H_2_O)_2_][BF_4_]_2_	
C1–N5–C17	119.67(13)	C1–N3–C9	113.56(11)
C1–N5–C18	120.65(12)	C1–N3–C10	115.43(11)
C14–N6–C19	114.75(11)	N3···O1	2.8692(16)
C14–N6–C20	110.71(11)		
N6···O1	2.6530(16)		
N5···N7	3.1282(17)		

The solid-state structures reveal hydrogen-bonding interactions between the aqua ligands and the dimethylamine functionalities. In [CoL^DMA^(CH_3_CN)(H_2_O)][BF_4_]_2_, the distance between the dimethylamine nitrogen (N6) and aqua oxygen (O1) is 2.6530(16) Å, compared to 3.1282(17) Å between the other dimethylamine nitrogen (N5) and nitrogen on the adjacent acetonitrile ligand (N7). For [CoL^DMA^(H_2_O)_2_][BF_4_]_2_, the structure was generated from a two-fold C2 axis; only one of the two hydrogen bonding interactions between the aqua ligands and dimethylamines are crystallographically unique. The distance between the dimethylamine nitrogen (N3) to oxygen (O1) on the aqua ligand is 2.8692(16) Å. The distances observed between the hydrogen bond acceptor and donor are in the range of a moderate hydrogen-bonding interaction.^38^

The hydrogen-bonding interactions are also evident from the geometry at the dimethylamines associated with aqua ligands compared to those with no exogenous interactions (Table 1). In [CoL^DMA^(CH_3_CN)(H_2_O)][BF_4_]_2_, the C(pyridine)–N(dimethylamine)–CH_3_ angles for N5, which does not participate in hydrogen-bonding, are 119.67(13)° and 120.65(12)°, which are expected for a tertiary amine. In contrast, N6, which is poised over the aqua ligand, has more acute angles, 114.75(11)° and 110.71(11)°, indicating a contraction to accommodate the hydrogen-bonding interaction with a proton from water. The bis-aqua bound structure [CoL^DMA^(H_2_O)_2_][BF_4_]_2_ also demonstrates angular distortion with acute N3 angles of 113.56(11)° and 115.43(11)°. Complete information regarding the refinement of each structure, as well as tables for all bond angles and distances can be found in the ESI (Fig. S16–S19, Tables S1–S7†).

### Electrochemical studies

**Fig. 2:**
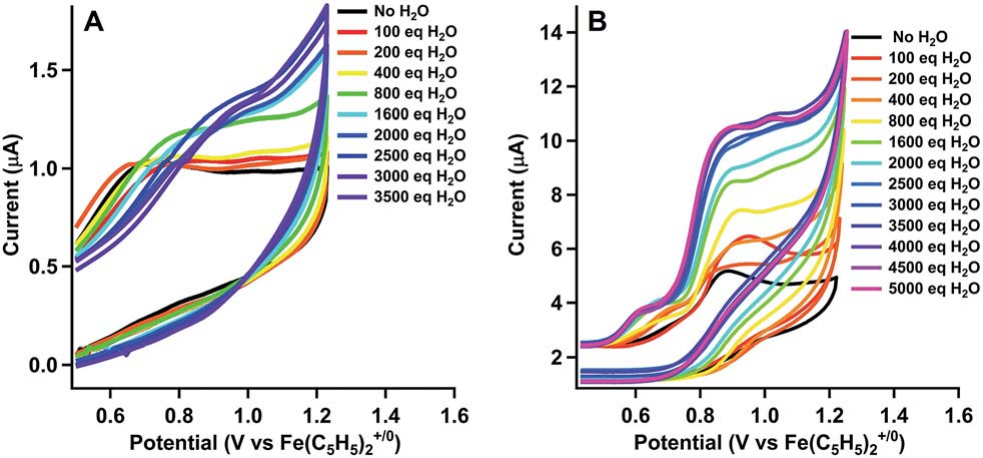
Cyclic voltammograms upon addition of water to 1 mM solutions of (a) [CoL^H^(CH_3_CN)_2_][BF_4_]_2_ (**1**) and (b) [CoL^DMA^(CH_3_CN)_2_][BF_4_]_2_ (**2**) in CH_3_CN at 100 mV s^–1^.

The cyclic voltammograms for the oxidation of [CoL^H^(CH_3_CN)_2_][BF_4_]_2_ (**1**) and [CoL^DMA^(CH_3_CN)_2_][BF_4_]_2_ (**2**) are shown as the black trace in Fig. 2a and b, respectively. Complex **1** and **2** exhibit an oxidation at *E*_pa_ = 0.77 V and 0.96 V *versus* Fe(C_5_H_5_)_2_^+/0^, respectively. Both oxidations are irreversible at scan rates up to 1600 mV s^–1^ (Fig. S4†). Cyclic voltammograms of **1** and **2** over a larger potential window are shown in Fig. S3.†

We tested activity for electrochemical water oxidation by adding aliquots of water to **1** and **2** in acetonitrile since the cobalt complexes are minimally soluble in water. The resulting cyclic voltammograms are shown as coloured traces in Fig. 2. The oxidative current for [CoL^H^(CH_3_CN)_2_][BF_4_]_2_ (**1**), which contains no pendant amines in the secondary coordination sphere, displayed minimal changes upon addition of up to 400 equivalents of water. A small current increase is observed with 400 to 3500 equivalents of water which does not appear to be due to water oxidation since negligible O_2_ is detected under controlled potential electrolysis at 1.07 V *vs.* Fe(C_5_H_5_)_2_^+/0^ (*vide infra*). The mild current increase may be due to catalytic hydration of acetonitrile to acetamide, a reaction that has been observed for similar Co(iii) complexes.^39^ Addition of between 3500 up to 5000 equivalents of water resulted in loss of the oxidation event. Full cyclic voltammetry data for [CoL^H^(CH_3_CN)_2_][BF_4_]_2_ (**1**) and water titration can be seen in Fig. S5.†

In contrast, [CoL^DMA^(CH_3_CN)_2_][BF_4_]_2_ (**2**) displayed an enhanced current response with increasing water content (up to 4000 equivalents), leading to a plateau profile characteristic of electrocatalytic activity where the catalyst is operating under pseudo-first order conditions.^40^ A negative shift in the electrochemical potential appears with increasing concentrations of water, which may be due to displacement of one or both acetonitrile ligands with aqua ligands. The magnitude of the plateau current increased linearly with the concentration of **2**, indicating the oxidation reaction is first order in **2** (Fig. S7 and S8†).

We also compared the electrochemical oxidation of **2** in the presence of D_2_O. Increasing equivalents of D_2_O leads to a similar current enhancement and plateau (see Fig. S6†). However, the current ceases to increase after addition of 800 equivalents. An accurate determination of the rate of water oxidation using cyclic voltammetry is challenging because the oxidation of **2** is not reversible under non-catalytic conditions. However the ratio of the current enhancement *vs.* the non-catalytic current decreases from 4 to 2.4 with H_2_O *vs.* D_2_O as substrate, indicating a 2-fold decrease in rate for a 4 electron process.^41^

**Fig. 3:**
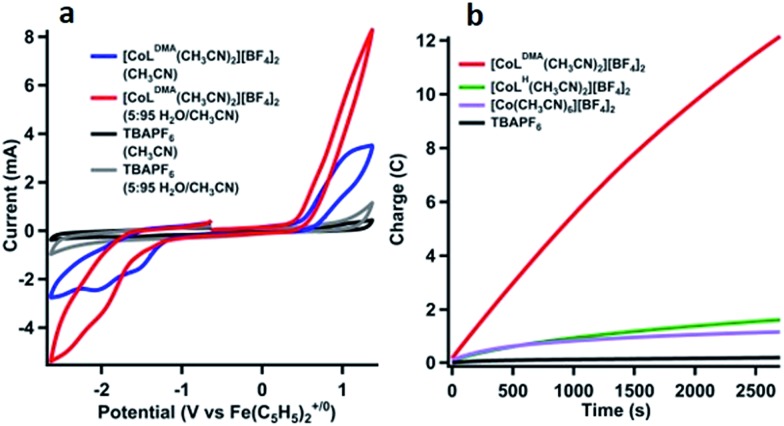
(a) Cyclic voltammograms in CPE cell. [CoL^DMA^(CH_3_CN)_2_][BF_4_]_2_ in 5 : 95 H_2_O/CH_3_CN (red), [CoL^DMA^(CH_3_CN)_2_][BF_4_]_2_ in 0 : 100 H_2_O/CH_3_CN (blue), and no catalyst present under the same conditions (gray and black). (b) Charge passed *vs.* time in the controlled potential electrolysis of a 1 mM solution of [CoL^DMA^(CH_3_CN)_2_][BF_4_]_2_ (**2**) in 95 : 5 CH_3_CN : H_2_O (v/v) at 1.07 V *vs.* Fe(C_5_H_5_)_2_^+/0^ (red trace). Equivalent electrolyses under the same conditions; [CoL^H^(CH_3_CN)_2_][BF_4_]_2_ (**1**, green trace), [Co(CH_3_CN)_6_][BF_4_]_2_ (lavender trace), and rinsed post-electrolysis electrode in fresh Bu_4_NPF_6_ solution (black trace).

The product of oxidation in the presence of water with [CoL^DMA^(CH_3_CN)_2_][BF_4_]_2_ (**2**) was identified using controlled potential electrolyses (CPE) and analysis of the headspace by gas chromatography.^42^Fig. 3a shows the cyclic voltammetry of 1 mM solutions of [CoL^DMA^(CH_3_CN)_2_][BF_4_]_2_ (**2**) prior to electrolysis in acetonitrile (blue trace) and in 95 : 5 CH_3_CN : H_2_O (v/v, red trace). The cyclic voltammograms with no compound present are shown in black (acetonitrile) and grey (95 : 5 CH_3_CN : H_2_O, v/v) in Fig. 3a, demonstrating no appreciable background current in the potential window under these conditions. Cyclic voltammetry with [CoL^H^(CH_3_CN)_2_][BF_4_]_2_ (**1**) under equivalent conditions in the controlled potential electrolysis cell are shown in Fig. S9.†

Charge passed during controlled potential electrolysis at 1.07 V *vs.* Fe(C_5_H_5_)_2_^+/0^ for 1 mM [CoL^DMA^(CH_3_CN)_2_][BF_4_]_2_ (**2**) in 95 : 5 CH_3_CN : H_2_O (v/v) is shown as the red trace in Fig. 3b. By comparison, a low current response is observed for [CoL^H^(CH_3_CN)_2_][BF_4_]_2_ (**1**) (green trace, Fig. 3b), which lacks pendant bases.

Analysis of the O_2_ concentration in the headspace after electrolysis of [CoL^DMA^(CH_3_CN)_2_][BF_4_]_2_ (**2**) (Fig. S11†) leads to a faradic yield of 99% for the oxidation of water to oxygen (∼1 TON). Longer electrolyses result in a decline of the faradic yield of O_2_. Electronic absorption spectra taken before and after electrolysis reveal changes in solution composition (Fig. S10†). These changes, coupled with a gradual decrease in current (Fig. 3b & S10†) and faradic yield under extended electrolysis, suggests degradation of **2** under oxidative conditions. Previous studies with ligands of this type have shown long-term instability under oxidizing conditions, with evidence of demetallation and ligand decomposition.^26^,^43^,^44^ Acetonitrile may also be susceptible to oxidative degradation under these conditions. An infrared spectrum of the post-electrolysis solution after solvent removal exhibits a small peak at 2060 cm^–1^, a value similar to cobalt cyanide complexes.^45^ (see Fig. S11†) Another contribution to the decline in activity may be an increase in solution acidity during electrolysis that promotes decomposition by ligand deprotonation. Studies have been performed on water oxidation in buffered mixed organic-aqueous solutions.^46^ However we found aqueous buffers (phosphate, carbonate) were insufficiently soluble in our solution and soluble organic bases (primarily amines) were oxidized at potentials negative of **2**.

The concentration-dependent oxidation activity (measured by the plateau current in the CV) indicates the active species is likely **2** (Fig. S7 and S8†). However, prior studies have shown that Co(ii) ions can leach into solution from molecular species to form active heterogeneous electrocatalysts in aqueous phosphate buffer.^47^ To minimize this possibility, we performed several additional experiments that support homogeneous water oxidation activity by **2**. An electrolysis of 1 mM solution of [Co(CH_3_CN)_6_][BF_4_]_2_ under identical conditions passed minimal current (lavender trace, Fig. 3b). For both [CoL^H^(CH_3_CN)_2_][BF_4_]_2_ (**1**) and [Co(CH_3_CN)_6_][BF_4_]_2_ (green and lavender trace, respectively, in Fig. 3b), the amount of oxygen in the headspace was below the detectable limit of our GC calibration curve, or less than 0.1%. All electrolyses were also performed with a mercury pool in the electrolysis cell to form an amalgam with potential cobalt oxide heterogeneous particles forming *in situ*.^47^,^48^ Additionally, the working electrode was rinsed with water and acetonitrile and reused in a fresh solution of 5% v/v solution of H_2_O in CH_3_CN to confirm no heterogeneous cobalt compounds had deposited onto the electrode surface (Fig. 3b, black trace). Due to sample size constraints we are unable to perform an XPS on the high-surface area vitreous carbon working electrode used to quantify O_2_ after controlled potential electrolysis. We instead performed an identical electrolysis using a smaller glassy carbon electrode. The current *vs.* time profile is similar to that observed with vitreous glassy carbon (Fig. S14†). No evidence of cobalt or cobalt oxide was observed in the XPS spectrum (Fig. S15†). Although it is difficult to completely rule out any contributions to water oxidation by heterogeneous species, the lack of induction period also suggests the initial species is evolving oxygen, and the decrease in activity indicates degradation into an inactive species.

The slightly higher reduction potential of [CoL^DMA^(CH_3_CN)_2_][BF_4_]_2_ (**2**) compared to [CoL^H^(CH_3_CN)_2_][BF_4_]_2_ (**1**) could potentially contribute to the difference in activity between the two complexes. However, this effect is minimal at higher concentrations of water since the onset potential for **2** decreases to roughly the same potential as **1** while maintaining an enhanced current response.

The water oxidation activity of **2***vs.***1** is more likely enabled by the presence of the pendant bases. It is possible the pendant bases facilitate catalysis by providing a favourable H_2_O binding environment through the hydrogen-bonding interactions. However, the p*K*_b_ and proximity of dimethylamine proton acceptors in the secondary coordination sphere are also appropriate for supporting concerted proton transfer. The p*K*_a_'s of related N-coordinated Co(ii) aqua complexes are between 6 and 8.^49^ Upon oxidation, the p*K*_a_ of metal aqua complexes typically increase by at least 6 units.^16^,^50^ We expect the dimethylamine pendant bases in L^DMA^ to have a similar p*K*_a_ to protonated dimethylaniline (5.07).^51^ The pendant bases are thus matched to accept a proton from an oxidized aqua complex and circumvent formation of a highly acidic (and energetic) intermediate.^6^,^7^,^26^,^52^–^57^ Hydrogen-bond acceptors have also been shown to stabilize cobalt hydroxide or hydroperoxo ligands.^58^,^59^ Our efforts to isolate or spectroscopically identify intermediates during water oxidation using stoichiometric chemical oxidants and bases were unsuccessful. However, the marked difference in water oxidation activity between **1** and **2**, along with the deuterium isotope effect, indicate the potential role of intramolecular proton acceptors in facilitating redox catalysis.

## Conclusions

The oxidation of water to oxygen is the vital half reaction to carbon dioxide reduction in photosynthesis. As a result, synthetic catalysts for this reaction are key to artificial photosynthetic schemes. We establish that appropriately positioned pendant bases can enable water oxidation activity in this system. Although the overall stability is an issue with this ligand set, our study underscores design principles for synthetic microenvironments that can be incorporated into more oxidatively stable catalysts.

## Conflicts of interest

There are no conflicts to declare.

## Supplementary Material

Supplementary information

Crystal structure data
