# Supplementary material for: Intramolecular hydrogen-bonding in a cobalt aqua complex and electrochemical water oxidation activity
Source: Chem Sci. 2018 Feb 6;9(10):2750–5. doi: 10.1039/c7sc04960a (PMC5912104; doi:10.1039/c7sc04960a)
Supplement: Supplementary file 1 [file SC-009-C7SC04960A-s001.pdf]

Electronic Supplementary Material (ESI) for Chemical Science.
This journal is © The Royal Society of Chemistry 2018

Intramolecular Hydrogen-Bonding in a Cobalt Aquo Complex and Electrochemical Water Oxidation Activity

Juliet F. Khosrowabadi Kotyk, Caitlin M. Hanna,
Rebecca L. Combs, Joseph W. Ziller, and Jenny Y. Yang*

Department of Chemistry, University of California, Irvine

Supplementary Information

Table of Contents

Experimental Details

General Experimental Details S3

Physical Methods (NMR Spectroscopy, Mass Spectrometry, UV-Visible Spectroscopy, EPR Spectroscopy, Elemental Analysis, Cyclic Voltammetry, Controlled Potential Electrolysis, Gas Chromatography, X-Ray Photoelectron Spectroscopy, X-Ray Crystallography S3

Synthetic Procedures

[CoL^{H}(CH$_3$CN)$_2$][BF$_4$]$_2$ (1) S5

[CoL^{DMA}(CH$_3$CN)$_2$][BF$_4$]$_2$ (2) S5

[CoL^{DMA}(CH$_3$CN)(H$_2$O)][BF$_4$]$_2$ S5

[CoL^{DMA}(H$_2$O)$_2$][BF$_4$]$_2$ S5

Physical Characterization

Figure S1. UV-Vis spectra of 5 μM CH$_3$CN solutions of [CoL^{H}(CH$_3$CN)$_2$][BF$_4$]$_2$ (**1**, black trace) and [CoL^{DMA}(CH$_3$CN)$_2$][BF$_4$]$_2$ (**2**, gray trace). Inset: UV-Vis spectra of 5 mM CH$_3$CN solutions, *d-d* transitions. S6

Figure S2. EPR spectra of [CoL^{H}(CH$_3$CN)$_2$][BF$_4$]$_2$ (**1**, black trace) and [CoL^{DMA}(CH$_3$CN)$_2$][BF$_4$]$_2$ (**2**, gray trace). All samples taken as 10 mM frozen CH$_3$CN solutions at 10 K. S7

Electrochemical Data

Figure S3. Cyclic voltammograms of 1 mM solutions of [CoL^{H}(CH$_3$CN)$_2$][BF$_4$]$_2$ (**1**, top, black) and [CoL^{DMA}(CH$_3$CN)$_2$][BF$_4$]$_2$ (**2**, bottom, gray) in 0.10 M Bu$_4$NBF$_4$ in CH$_3$CN at 100 mV/s. S8

Figure S4. Scan rate dependent cyclic voltammograms of the Co(II/III) oxidation events of 1.0 mM solutions of A) [CoL^{H}(CH$_3$CN)$_2$][BF$_4$]$_2$ (**1**) and B) [CoL^{DMA}(CH$_3$CN)$_2$][BF$_4$]$_2$ (**2**) in 0.10 M Bu$_4$NBF$_4$ in CH$_3$CN at scan rates 25–1600 mV/s. S8

Figure S5. Cyclic voltammograms upon addition of water to 1 mM solutions of [CoL^{H}(CH$_3$CN)$_2$][BF$_4$]$_2$ (**1**) in CH$_3$CN at 100 mv/s. S9

Figure S6. Cyclic voltammograms upon addition of D$_2$O to 1.0 mM solutions of [CoL^{DMA}(CH$_3$CN)$_2$][BF$_4$]$_2$ (**2**) in 0.10 M Bu$_4$NBF$_4$ in CH$_3$CN at 100 mv/s. S9

Figure S7. Cyclic voltammograms at various concentrations of [CoL^{DMA}(CH$_3$CN)$_2$][BF$_4$]$_2$ (**2**) in 0.10 M Bu$_4$NBF$_4$ in 5:95 H$_2$O/CH$_3$CN at 100 mv/s. S10

Figure S8. Peak current observed by cyclic voltammetry vs concentration of [$CoL^{DMA}(CH_3CN)_2$][BF_4]$_2$ (**2**) in 0.10 M Bu_4NBF_4 in 5:95 H_2O/CH_3CN at 100 mv/s. Inset contains numbers used for graph. S10

Controlled Potential Electrolysis and Gas Chromotography

Figure S9. Cyclic voltammograms in controlled potential electrolysis cell. [$CoL^{H}(CH_3CN)_2$][BF_4]$_2$ (**1**) in 5:95 H_2O/CH_3CN (red) and 0:100 H_2O/CH_3CN (blue) and no catalyst present under the same conditions (gray and black). S11

Figure S10. Current vs time plot of 45 minutes electrolysis of 1 mM solution of [$CoL^{DMA}(CH_3CN)_2$][BF_4]$_2$ (**2**) in 95:5 CH_3CN:H_2O (v/v) at 1.07 V vs $Fe(C_5H_5)_2^{+/0}$. S11

Figure S11. A) GC calibration curve for O_2. Points show the average peak area and error bars show the standard deviation across five measurements. Linear fit equation is shown on graph. B) Representative GC trace for headspace of [$CoL^{DMA}(CH_3CN)_2$][BF_4]$_2$ (**2**) electrolysis. S12

Figure S12. UV-Vis spectra A) before (black trace) and after (dark gray trace) CPE, 1 mM solution of [$CoL^{DMA}(CH_3CN)_2$][BF_4]$_2$ (**2**) in 95:5 CH_3CN:H_2O (v/v). S12

Figure S13. Solid-state infrared spectra of [$CoL^{DMA}(CH_3CN)_2$][BF_4]$_2$ (**2**) (red trace) and of post CPE solution (from conditions described in experimental) upon solvent removal. S13

Figure S14. (Top) Current vs time plot and (bottom) charge vs time plot (total 1.66 C) for 30 minutes of controlled potential electrolysis (CPE) of 1 mM solution of [$CoL^{DMA}(CH_3CN)_2$][BF_4]$_2$ (**2**) in 95:5 CH_3CN:H_2O (v/v) at 1.07 V vs $Fe(C_5H_5)_2^{+/0}$ using a glassy carbon electrode. The electrode was removed after this experiment and its XPS spectra is shown in Figure S13. S14

Figures S15. X-Ray Photoelectron Spectroscopy of glassy carbon electrode post electrolysis. (Top) Survey spectrum and (bottom) high-resolution Co 2p region. S15

Crystallographic Data

Figure S16. ORTEP of [$CoL^{H}(CH_3CN)_2$][BF_4]$_2$ (**1**) showing atomic numbering scheme. S16

Table S1. Crystal data and structure refinement for [$CoL^{H}(CH_3CN)_2$][BF_4]$_2$ (**1**). S16

Table S2. Bond lengths [Å] and angles [°] for [$CoL^{H}(CH_3CN)_2$][BF_4]$_2$ (**1**). S17

Figure S17. Ball and stick depiction of [$CoL^{DMA}(CH_3CN)_2$][BF_4]$_2$ (**2**) showing atomic numbering scheme. S19

Table S3. Crystal data and structure refinement for [$CoL^{DMA}(CH_3CN)_2$][BF_4]$_2$ (**2**). No metrical parameters are provided due to the poor quality of the data set; the structure is reported for connectivity information only. S20

Figure S18. ORTEP of [$CoL^{DMA}(CH_3CN)(H_2O)$][BF_4]$_2$ showing atomic numbering scheme. S21

Table S4. Crystal data and structure refinement for [$CoL^{DMA}(CH_3CN)(H_2O)$][BF_4]$_2$ S21

Table S5. Bond lengths [Å] and angles [°] for [$CoL^{DMA}(CH_3CN)(H_2O)$][BF_4]$_2$. S22

Figure S19. ORTEP of [$CoL^{DMA}(H_2O)_2$][BF_4]$_2$ showing atomic numbering scheme. S24

Table S6. Crystal data and structure refinement for [$CoL^{DMA}(H_2O)_2$][BF_4]$_2$. S25

Table S7. Bond lengths [Å] and angles [°] for [$CoL^{DMA}(H_2O)_2$][BF_4]$_2$. S25

References S27

Experimental Details

General Experiment Considerations

All reagents were purchased from commercial suppliers and used without purification. Unless otherwise noted, all organic chemical manipulations were performed in air. Compounds were purified via flash column chromatography using Sorbent Technologies 60 Å, 230–400 mesh silica gel, unless otherwise stated. Unless otherwise noted, inorganic metal syntheses were performed in a Vacuum Atmospheres Co. drybox under a nitrogen atmosphere. $[Co(CH_3CN)_6][BF_4]_2$ was prepared using literature methods.[1] L^H and L^{DMA} were synthesized as previously described.[2] Anhydrous solvents were sparged with UHP argon (Praxair) and passed through columns containing Q-5 and molecular sieves before use.

Physical Methods

Nuclear Magnetic Resonance (NMR) Spectroscopy: ^1H NMR spectra were recorded on 500 MHz on Bruker instruments. ^1H NMR spectra chemical shifts are reported as *δ* values in ppm relative to residual protio solvent: $CDCl_3$ (7.26 ppm), CD_3CN (1.94 ppm). Proton NMR data are reported as follows: chemical shift (*δ* ppm), multiplicity (s = singlet, d = doublet, t = triplet, q = quartet), coupling constants (*J*) in Hertz (Hz), and integration. Multiplets (m) are reported over the range (ppm). Data for ^{13}C NMR spectra are decoupled proton spectra and are reported in terms of chemical shift (*δ* ppm).

Mass Spectrometry (MS): High resolution mass spectra (HR-MS) and electrospray ionization mass spectra (ESI-MS) were obtained on a Micromass LCT and collected at the University of California-Irvine Mass Spectrometry Facility.

Ultraviolet-visible (UV-Vis) Spectroscopy: Ultraviolet-visible spectra were collected as 10 µM solutions in 3 mL CH_3CN in a 1 cm quartz cuvette or as 1mM solutions in 0.3 mL CH_3CN in a 1 mm quartz cuvette using an Agilent Technologies Cary 60 UV–vis spectrometer.

Electron Paramagnetic Resonance (EPR) Spectroscopy: X-band (9.28 GHz) EPR spectra were collected as 10 mM frozen solutions using a Bruker EMX spectrometer equipped with an ER041XG microwave bridge at 77 K or 10 K.

Elemental Analysis (EA): Elemental analyses were performed on a Perkin Elmer 2400 Series II CHNS elemental analyzer.

Cyclic Voltammetry (CV): Electrochemical experiments were performed under an atmosphere of nitrogen in a solution containing 0.1 M Bu_4NBF_4 or Bu_4NPF_6 in acetonitrile. Glassy carbon was used as the working and auxiliary electrode and a silver wire was used as a pseudoreference electrode. Ferrocene was used as an internal standard, and all potentials are referenced to $Fe(C_5H_5)_2^{+/0}$. Cyclic voltammetry experiments were performed with a Pine Wavedriver 10 or 20 potentiostat and Pine Aftermath software version 1.4.7881.

Controlled Potential Electrolysis:[3] Controlled potential electrolyses were performed in a Pine 150 mL cell for rotating electrodes with water jacket and five ground-glass ports (one 24/40, four 14/20). All cell ports were sealed with greased ground-glass joints with the exception of one 14/20 port, which was sealed with a SubaSeal rubber septum used for headspace sampling. Vitreous carbon foam rods were used for the working and counter electrodes, while an acetonitrile Ag/Ag^+ wire electrode was used as the reference. The carbon foam rods were attached to copper wire leads using conductive silver epoxy (AI Technology Prima-Solder EG8050) under Loctite epoxy. The counter electrode was separated from the bulk solution by a 10 mm glass tube with an extra-fine (Ace glass porosity E) fritted glass bottom; the tube was inserted

through the 24/40 cell port and sealed with a ground-glass joint. The working and reference electrodes were connected to the exterior of the cell via nickel sleeves joining the electrode leads to a tungsten wire that was sealed through a 14/20 ground-glass stopper. The potential values in the CPE were adjusted to be referenced versus $Fe(C_5H_5)_2^{+/0}$ by using E_{pa2} for 1.0 mM solution $[CoL^{DMA}(CH_3CN)_2][BF_4]_2$ in 0.10 M Bu_4NBF_4 in CH_3CN at 100 mv/s. A mercury pool, contained in a shallow glass cup, was placed at the bottom of each electrolysis sample. Cell headspace was sampled with a Restek A-2 Luer lock gas-tight syringe.

Gas Chromatography (GC): Headspace oxygen was quantified by gas chromatography on an Agilent 7890B instrument with a Molsieve column. GC method details are as follows: helium carrier gas, 40 °C column temperature, TCD detector at 200 °C. Calibration curves were generated by injecting known percentages of O_2 into a nitrogen filled round bottom flask (235.4 mL) and stirring for 10 mins. Background oxygen formation by the carbon foam electrode was estimated by running a controlled potential electrolysis of equal duration (45 min) on a blank electrolyte solution with no catalyst.

X-Ray Photoelectron Spectroscopy (XPS): Controlled potential electrolysis for XPS analysis of the working electrode was performed in a custom-made cell with two compartments separated by a fine glass frit. The working compartment contained 5 mL of 1 mM $[CoL^{DMA}(CH_3CN)_2][BF_4]_2$ and 100 mM Bu_4NPF_6 in 95:5 CH_3CN:H_2O, v/v, while the counter compartment contained 5 mL 100 mM Bu_4NPF_6 in CH_3CN. Both compartments were sealed with Silicone and Silicone/PTFE septa (Ace glass). Glassy carbon rods were used for the working and counter electrodes while an acetonitrile Ag/Ag^+ wire electrode was used as the reference. A mercury pool sat in a well at the bottom of the working compartment. Electrolysis was performed long enough to achieve one turnover after which the working electrode was removed and dried without rinsing for XPS analysis.

X-ray photoelectron spectra were acquired with a Kratos Analytical AXIS Supra spectrometer utilizing monochromatic Al Kα radiation (1486.7 eV, 250 W) under ultra-high vacuum (UHV) conditions ($\sim 10^{-9}$ Torr). Survey spectra were collected with a pass energy of 160 eV and high-resolution scans were performed with a pass energy of 20 eV. The binding energies were referred to the C 1s signal at 285 eV. Spectral data was analyzed using Computer Aided Surface Analysis for X-ray Photoelectron Spectroscopy (CasaXPS).

X-Ray Crystallography: A Bruker SMART APEX II diffractometer was used to collect all data. The APEX2 (APEX2 Version 2014.11-0, Bruker AXS, Inc.; Madison, WI 2014) program package was used to determine the unit-cell parameters and for data collection (20 sec/frame scan time for a sphere of diffraction data). The raw frame data was processed using SAINT (SAINT Version 8.34a, Bruker AXS, Inc.; Madison, WI 2013) and SADABS (Sheldrick, G. M. SADABS, Version 2014/5, Bruker AXS, Inc.; Madison, WI 2014) to yield the reflection data file. Subsequent calculations were carried out using the SHELXTL (Sheldrick, G. M. SHELXTL, Version 2014/7, Bruker AXS, Inc.; Madison, WI 2014) program. The structures were solved by direct methods and refined on F^2 by full-matrix least-squares techniques. The analytical scattering factors for neutral atoms were used throughout the analysis (International Tables for Crystallography 1992, Vol. C., Dordrecht: Kluwer Academic Publishers). The O-H hydrogen atoms on water of $[CoL^{DMA}(CH_3CN)(H_2O)][BF_4]_2$ and $[CoL^{DMA}(H_2O)_2][BF_4]_2$ were located from a difference-Fourier map and refined. The molecule $[CoL^{DMA}(H_2O)_2][BF_4]_2$ was located on a two-fold rotation axis. The remaining hydrogen atoms were included using a riding model.

Synthetic Procedures

[CoL^H(CH$_3$CN)$_2$][BF$_4$]$_2$ (1). [Co(CH$_3$CN)$_6$][BF$_4$]$_2$ (180 mg, 0.40 mmol) was added to a solution of **L^H** (109 mg, 0.40 mmol) in 5 mL of CH_3CN. The dark orange solution was stirred at 25 °C for 0.5 h upon which 5 mL of Et_2O was added and the precipitate isolated by filtration. The crude product, isolated as a brown precipitate, was redissolved in *ca.* 1 mL of CH_3CN and layered with 4 mL of Et_2O. After 1 day at −40 °C, the pure orange product was isolated by filtration and washed with 2 × 4 mL Et_2O. (170 mg, 78% yield). Single dark orange crystals suitable for X-ray crystallographic analysis were obtained by diffusion of Et_2O in a CH_3CN solution. ESI-MS (CH_3CN) *m/z*: 164.4 ($[CoL^H]^{2+}$). $\lambda_{max,}$ nm (CH_3CN, ε ($M^{-1} \times cm^{-1}$)) = 257 (4,000) and 465 (80). Anal. Calcd (Found) for $C_{16}H_{22}B_2CoF_8N_4$ (%): C, 38.21 (38.25); H, 4.41 (4.79); N, 11.14 (11.30).

[CoL^{DMA}(CH$_3$CN)$_2$][BF$_4$]$_2$ (2). [Co(CH$_3$CN)$_6$][BF$_4$]$_2$ (134 mg, 0.28 mmol) was added to a solution of **L^{DMA}** (103 mg, 0.128 mmol) in 5 mL of CH_3CN. The dark orange solution was stirred at 25 °C for 0.5 h and then 5 mL of Et_2O was added to precipitate the complex. The supernatant was decanted and resulting tan powder redissolved in *ca.* 1 mL of CH_3CN and layered with 4 mL of Et_2O. After 1 d at −40 °C, the dark orange product was isolated by filtration and washed with 2 × 4 mL Et_2O (170 mg, 89% yield). Single light orange crystals suitable for X-ray crystallographic analysis were obtained by diffusion of Et_2O in a CH_3CN solution. ESI-MS (CH_3CN) *m/z*: 207.7 ($[CoL^{DMA}]^{2+}$). $\lambda_{max,}$ nm (CH_3CN, ε ($M^{-1} \times cm^{-1}$)) = 256 (27,000), 324 (17,000) and 411 (130). Anal. Calcd (Found) for $C_{20}H_{32}B_2CoF_8N_6$ (%): C, 40.78 (40.40); H, 5.48 (5.43); N, 14.27 (14.39).

[CoL^{DMA}(CH$_3$CN)(H$_2$O)][BF$_4$]$_2$. H_2O (1.1 µL, 2 equiv) was added to a solution of [CoL^{DMA}(CH$_3$CN)$_2$][BF$_4$]$_2$ (20 mg, 0.03 mmol) in 5 mL of CH_3CN. The orange solution was stirred at 25 °C for 0.5 h and then 5 mL of Et_2O was added to precipitate the complex and was left overnight at −40 °C. The supernatant was decanted and resulting pink powder was isolated (8 mg, 40% yield). Removal of solvent resulted in an insoluble blue solid attributed to the loss of the aquo ligand. Single orange crystals suitable for X-ray crystallographic analysis were obtained by diffusion of Et_2O in a CH_3CN solution. APCI-MS (CH_3CN) *m/z*: 433.3 ($[CoL^{DMA} + H_2O]^+$).

[CoL^{DMA}(H$_2$O)$_2$][BF$_4$]$_2$. H_2O (1.1 µL, 2 equiv) was added to a solution of [CoL^{DMA}(CH$_3$CN)$_2$][BF$_4$]$_2$ (20 mg, 0.03 mmol) in 5 mL of DCM. The peach solution was stirred at 25 °C for 0.5 h. Removal of solvent resulted in an insoluble pink solid. Single dark pink crystals were obtained by diffusion of Et_2O in a DCM solution.

Physical Characterization

Electronic Absorption Spectroscopy

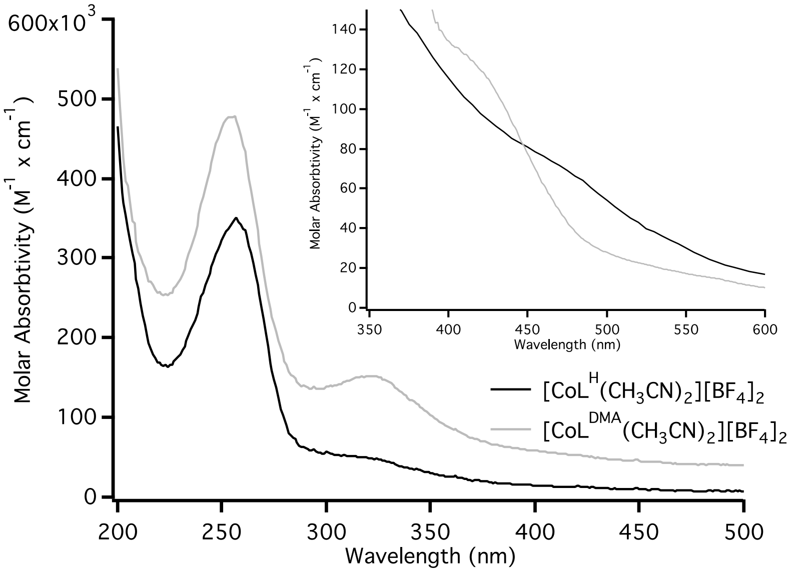

Figure S1. UV-Vis spectra of 5 μM CH_3CN solutions of $[CoL^H(CH_3CN)_2][BF_4]_2$ (**1**, black trace) and $[CoL^{DMA}(CH_3CN)_2][BF_4]_2$ (**2,** gray trace). Inset: UV-Vis spectra of 5 mM CH_3CN solutions, *d*-*d* transitions.

The UV-Vis profile of $[CoL^H(CH_3CN)_2][BF_4]_2$ (**1**) contains an absorption band at 257 nm which is attributed to the pyridyl π–π* transitions within the ligand. The shoulder at 465 nm is assigned to the *d*-*d* transition of the metal center which is typical of d^7 octahedral Co(II) complexes.[4]

$[CoL^{DMA}(CH_3CN)_2][BF_4]_2$ (**2**) contains two absorption bands due to two pyridyl π–π* transitions that occur at 256 and 324 nm, respectively. The secondary absorption band is indicative of the tautomeric structure possible for heteroatom substituted pyridines.[5] The *d*-*d* transition for the cobalt complex containing the L^{DMA} ligand occurs at 411 nm.

Electron Paramagnetic Resonance (EPR) Spectroscopy

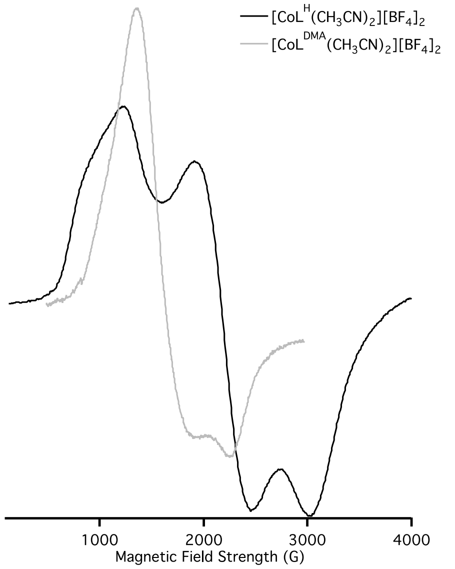

Figure S2. EPR spectra of $[CoL^{H}(CH_3CN)_2][BF_4]_2$ (**1**, black trace) and $[CoL^{DMA}(CH_3CN)_2][BF_4]_2$ (**2,** gray trace). All samples taken as 10 mM frozen CH_3CN solutions at 10 K.

Both cobalt complexes exhibit rhombic signals with g values typical of Co(II) high spin S=3/2 systems.[6] $[CoL^{H}(CH_3CN)_2][BF_4]_2$ (**1**) has g values of 5.65, 3.19, and 2.13. $[CoL^{DMA}(CH_3CN)_2][BF_4]_2$ (**2**) has g values of 4.19, and 2.66.

Electrochemical Data

Cyclic Voltammetry

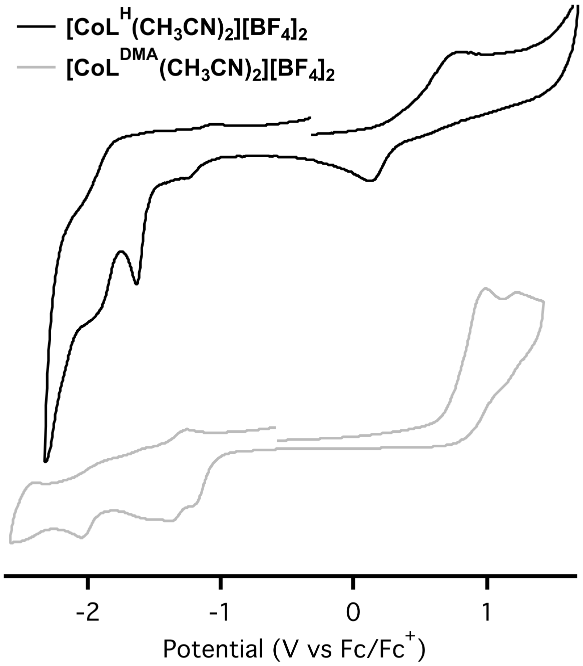

Figure S3. Cyclic voltammograms of 1 mM solutions of $[CoL^{H}(CH_3CN)_2][BF_4]_2$ (**1**, top, black) and $[CoL^{DMA}(CH_3CN)_2][BF_4]_2$ (**2**, bottom, gray) in 0.10 M Bu_4NBF_4 in CH_3CN at 100 mV/s. **1** exhibits an oxidation at E_{pa} = 0.77 V versus $Fe(C_5H_5)_2^{+/0}$. The two reduction events are observed at E_{pc} = −1.51 and −2.17 V, respectively. The oxidation of **2** occurs at E_{pa} = 0.96 V.

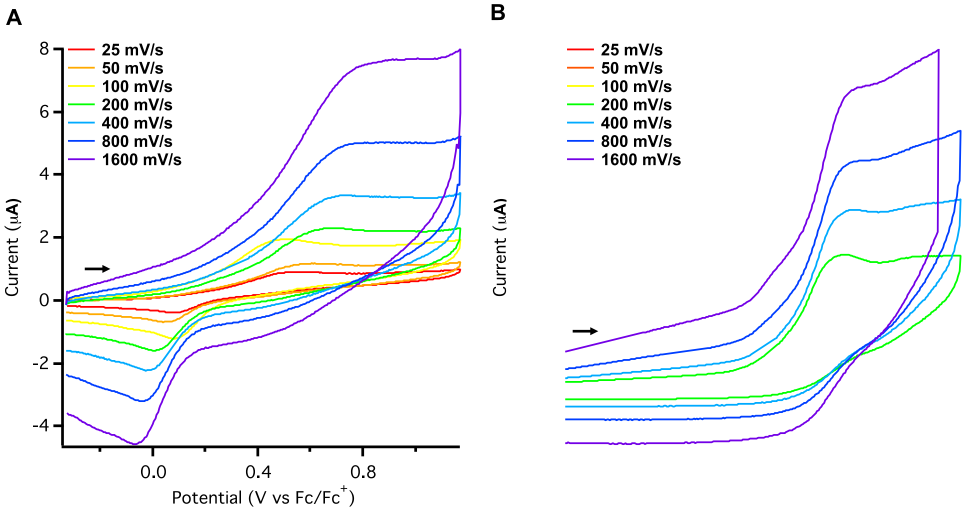

Figure S4. Scan rate dependent cyclic voltammograms of the Co(III/II) oxidation events of 1 mM solutions of A) $[CoL^{H}(CH_3CN)_2][BF_4]_2$ (**1**) and B) $[CoL^{DMA}(CH_3CN)_2][BF_4]_2$ (**2**) in 0.10 M Bu_4NBF_4 in CH_3CN at scan rates 25–1600 mV/s.

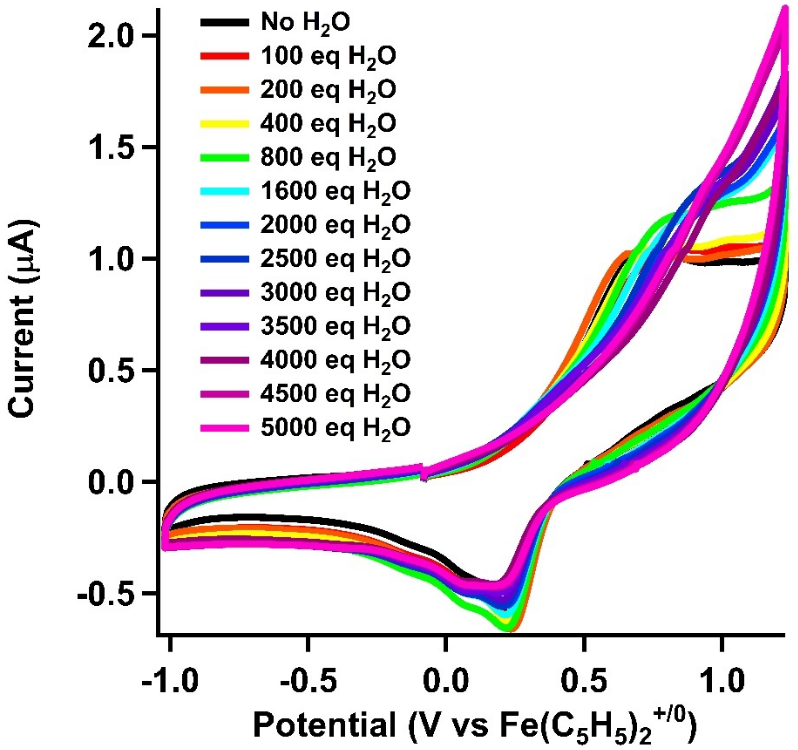

Figure S5. Cyclic voltammograms upon addition of water to 1 mM solutions of $[CoL^H(CH_3CN)_2][BF_4]_2$ (**1**) in CH_3CN at 100 mv/s.

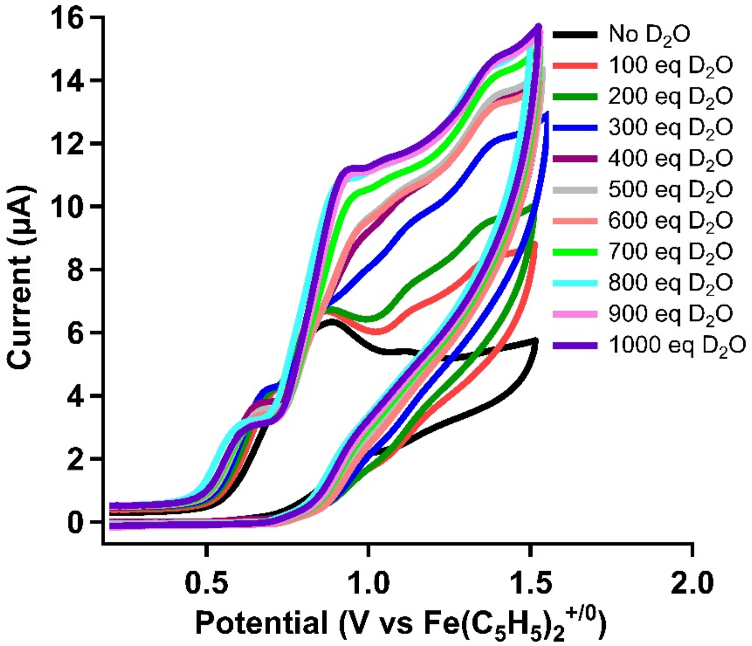

Figure S6. Cyclic voltammograms upon addition of D_2O to 1 mM solutions of $[CoL^{DMA}(CH_3CN)_2][BF_4]_2$ (**2**) in 0.10 M Bu_4NBF_4 in CH_3CN at 100 mv/s.

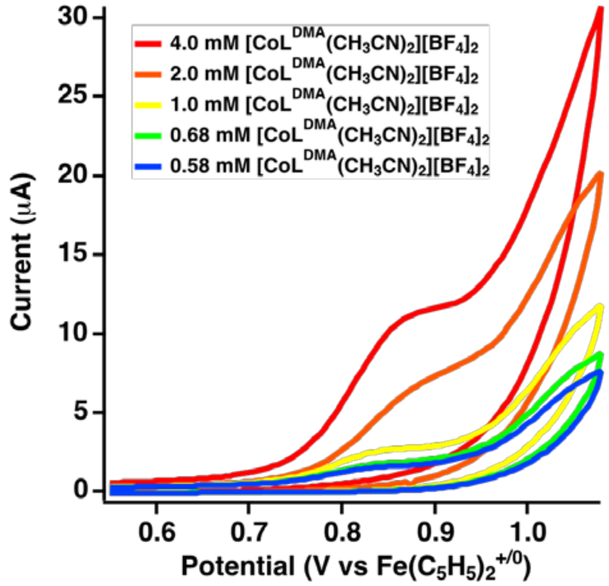

Figure S7. Cyclic voltammograms at various concentrations of $[CoL^{DMA}(CH_3CN)_2][BF_4]_2$ (**2**) in 0.10 M Bu_4NBF_4 in 5:95 H_2O/CH_3CN at 100 mv/s.

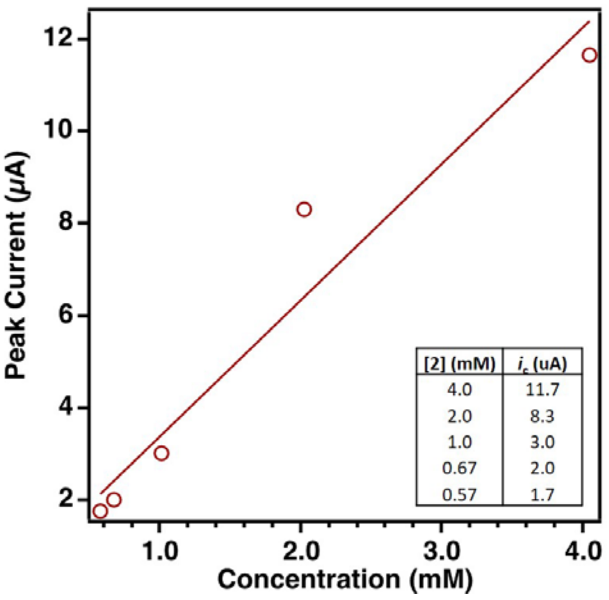

[2] (mM)	i_c (uA)
4.0	11.7
2.0	8.3
1.0	3.0
0.67	2.0
0.57	1.7

Figure S8. Peak current observed by cyclic voltammetry vs concentration of $[CoL^{DMA}(CH_3CN)_2][BF_4]_2$ (**2**) in 0.10 M Bu_4NBF_4 in 5:95 H_2O/CH_3CN at 100 mv/s. Inset contains numbers used for graph.

Controlled Potential Electrolysis and Gas Chromatography

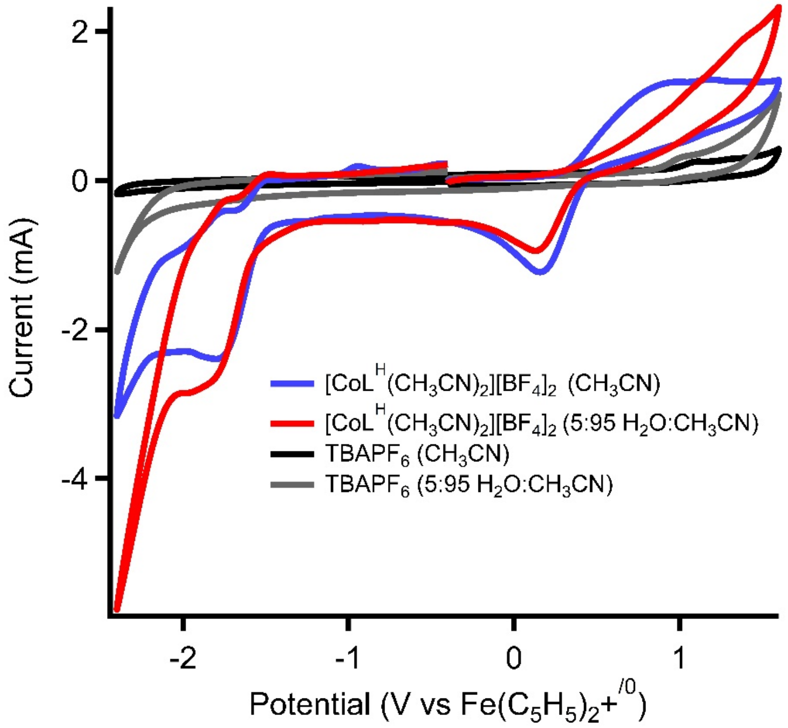

Figure S9. Cyclic voltammograms in controlled potential electrolysis cell. $[CoL^H(CH_3CN)_2][BF_4]_2$ (**1**) in 5:95 H_2O/CH_3CN (red) and 0:100 H_2O/CH_3CN (blue) and no catalyst present under the same conditions (gray and black).

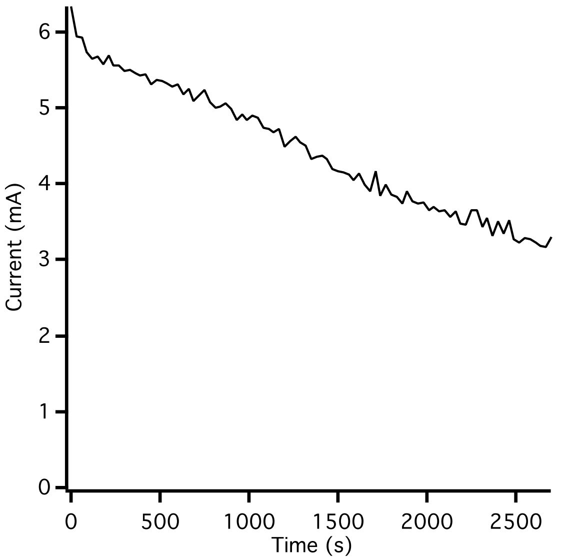

Figure S10. Current vs time plot of 45 minutes controlled potential electrolysis (CPE) of 1 mM solution of $[CoL^{DMA}(CH_3CN)_2][BF_4]_2$ (**2**) in 95:5 $CH_3CN:H_2O$ (v/v) at 1.07 V vs $Fe(C_5H_5)_2^{+/0}$ using a vitreous carbon electrode. The concentration of O_2 gas was quantified under these conditions.

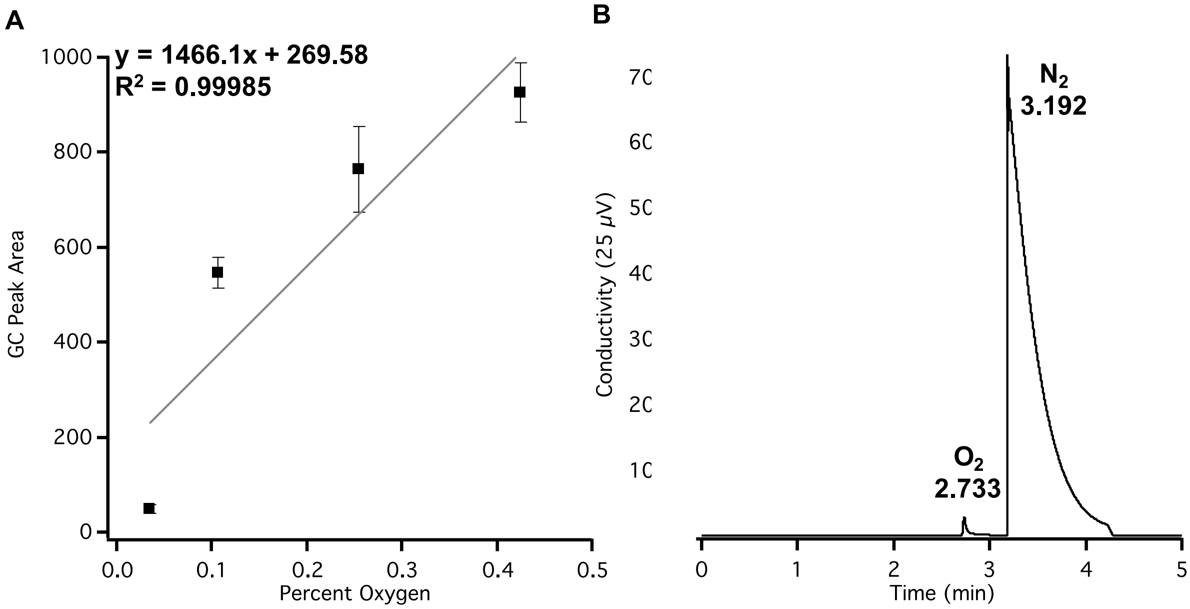

Figure S11. A) GC calibration curve for O_2. Points show the average peak area and error bars show the standard deviation across five measurements. Linear fit equation is shown on graph. B) Representative GC trace for headspace after 45 minutes of electrolysis of 1 mM solution of $[CoL^{DMA}(CH_3CN)_2][BF_4]_2$ (**2**) in 95:5 CH_3CN:H_2O (v/v) at 1.07 V vs $Fe(C_5H_5)_2^{+/0}$.

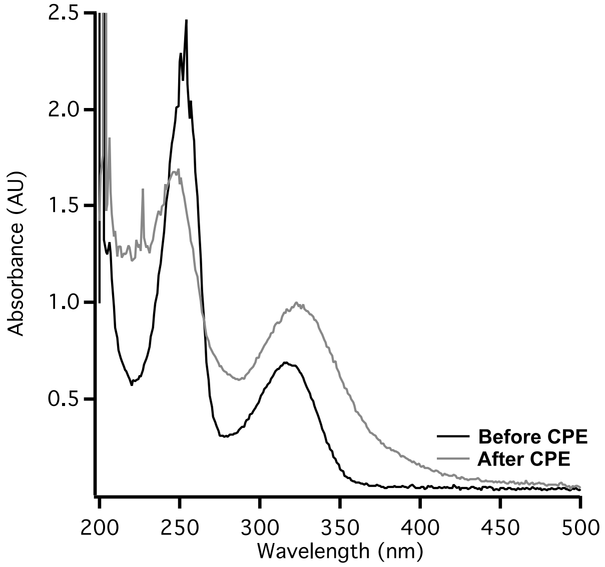

Figure S12. UV-Vis spectra A) before (black trace) and after (dark gray trace) CPE, 1 mM solution of $[CoL^{DMA}(CH_3CN)_2][BF_4]_2$ (**2**) in 95:5 CH_3CN:H_2O (v/v).

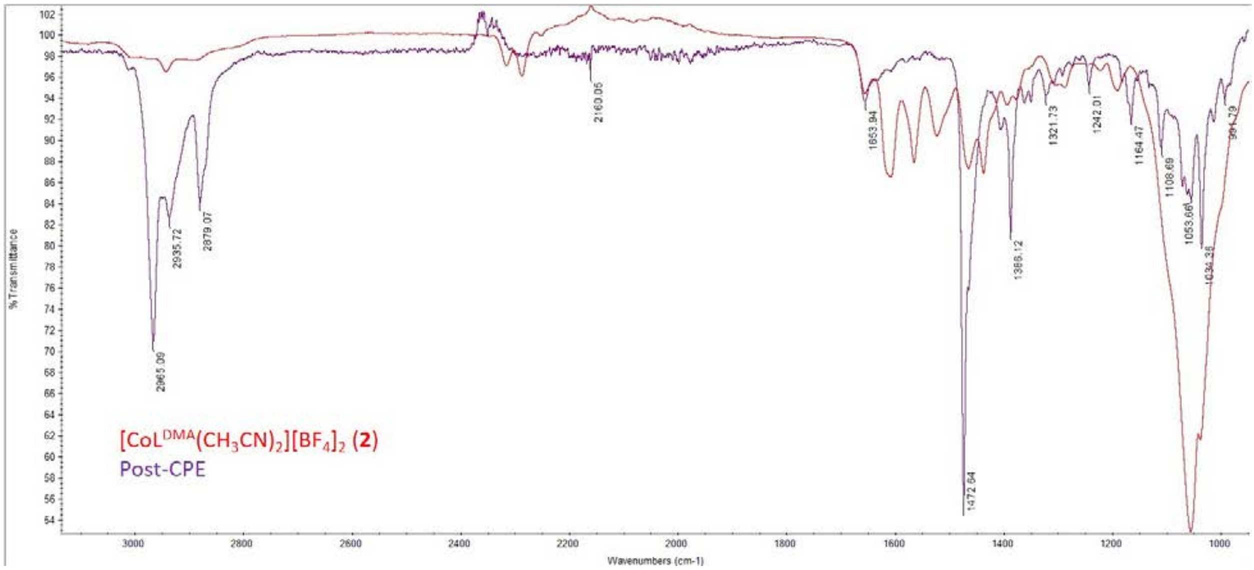

Figure S13. Solid-state infrared spectra of $[CoL^{DMA}(CH_3CN)_2][BF_4]_2$ (**2**) (red trace) and of post CPE solution (from conditions described in experimental) upon solvent removal.

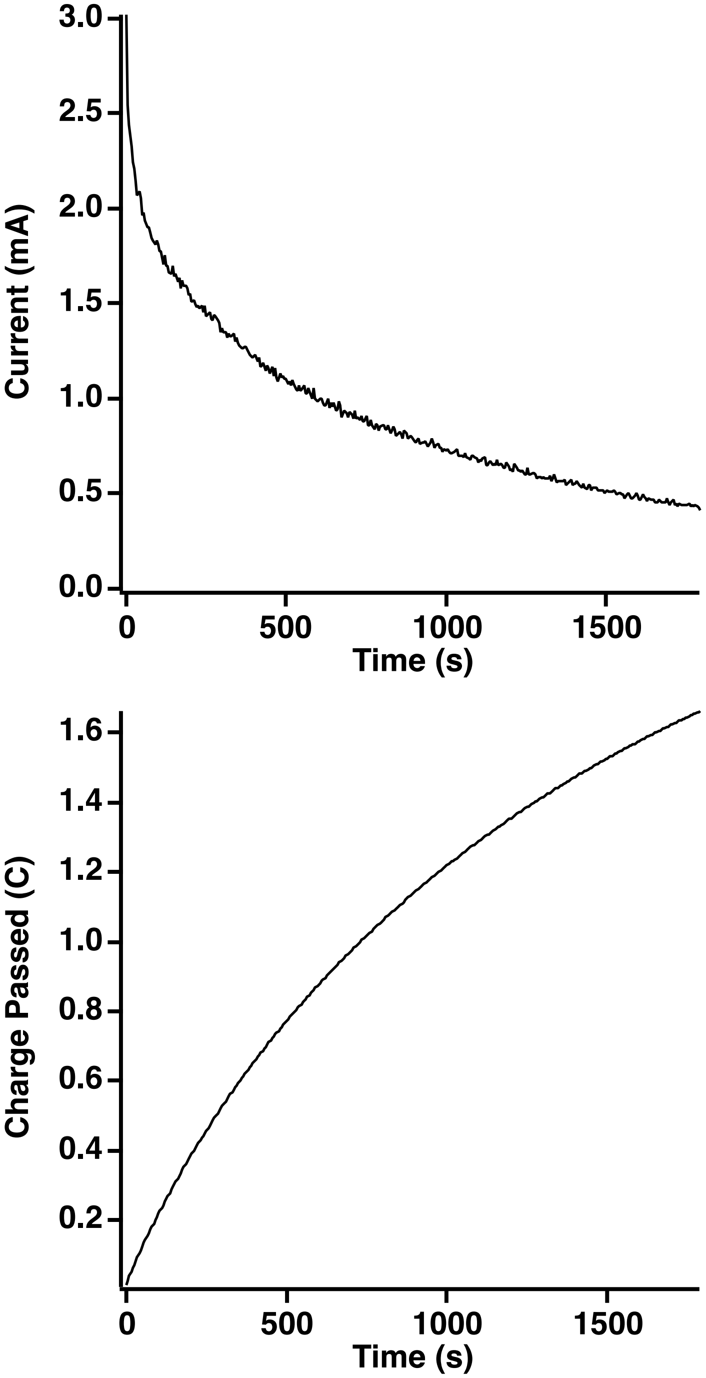

Figure S14. (Top) Current vs time plot and (bottom) charge vs time plot (total 1.66 C) for 30 minutes of controlled potential electrolysis (CPE) of 1 mM solution of $[CoL^{DMA}(CH_3CN)_2][BF_4]_2$ (**2**) in 95:5 CH_3CN:H_2O (v/v) at 1.07 V vs $Fe(C_5H_5)_2^{+/0}$ using a glassy carbon electrode. The electrode was removed after this experiment and its XPS spectra is shown in Figure S13.

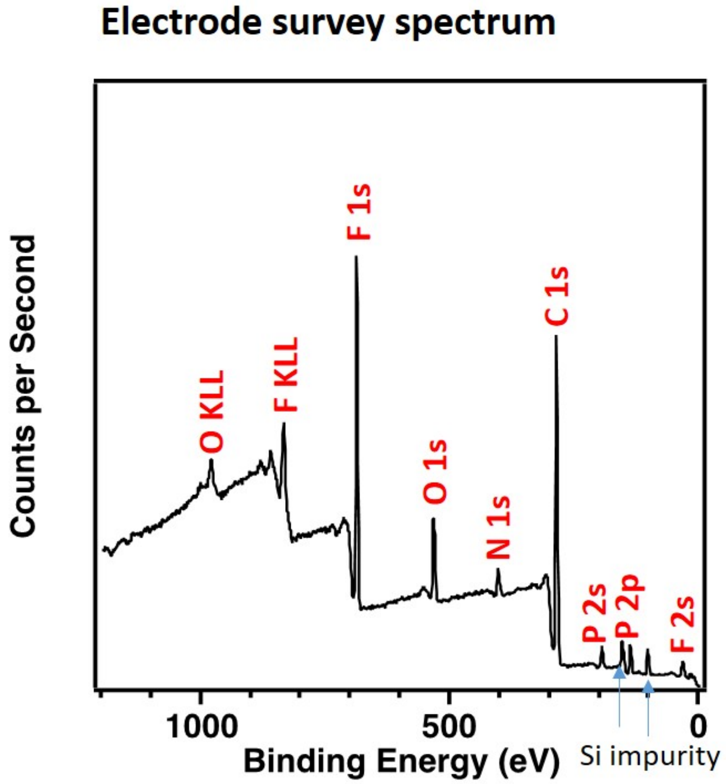

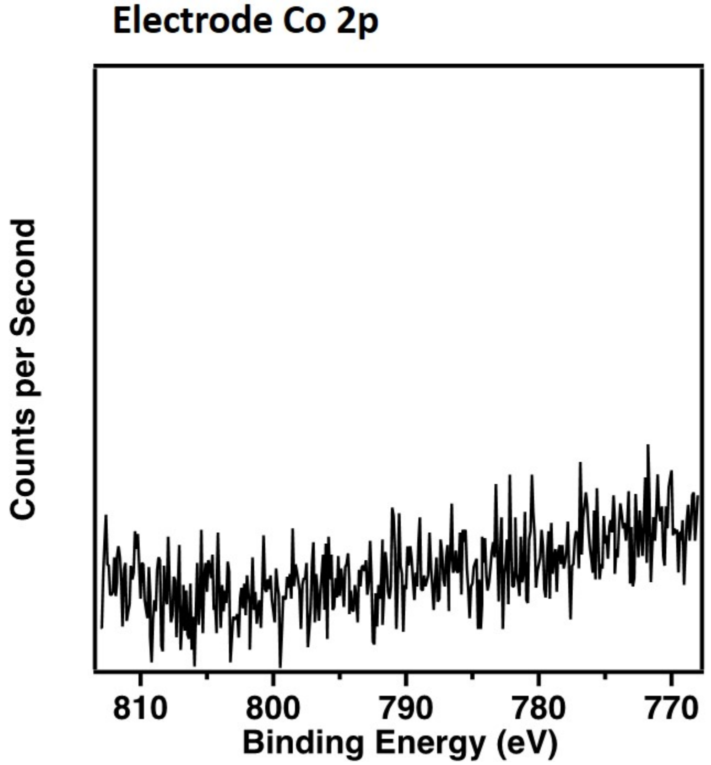

Figures S15. X-Ray Photoelectron Spectroscopy of glassy carbon electrode post electrolysis. (Top) Survey spectrum and (bottom) high-resolution Co 2p region.

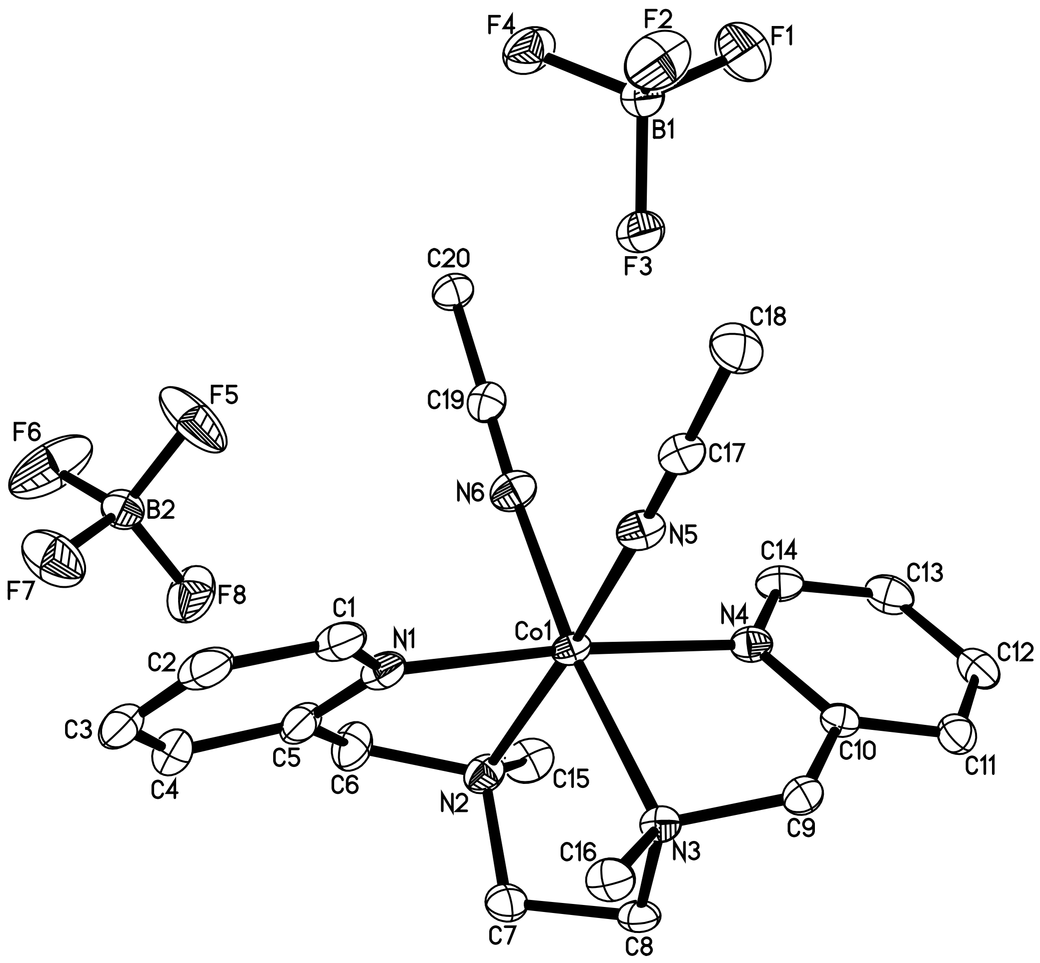

Figure S16. ORTEP of [CoL^H(CH$_3$CN)$_2$][BF$_4$]$_2$ (**1**) showing atomic numbering scheme.

Table S1. Crystal data and structure refinement for [CoL^H(CH$_3$CN)$_2$][BF$_4$]$_2$ (**1**).

Empirical formula	C_{20} H_{28} B_2 Co F_8 N_6	
Formula weight	585.03	
Temperature	88(2) K	
Wavelength	0.71073 Å	
Crystal system	Orthorhombic	
Space group	$P2_12_12_1$	
Unit cell dimensions	a = 9.8793(6) Å	α= 90°.
	b = 14.9748(8) Å	β= 90°.
	c = 17.3244(10) Å	γ = 90°.
Volume	2563.0(3) Å^3	
Z	4	
Density (calculated)	1.516 Mg/m^3	
Absorption coefficient	0.749 mm^{-1}	
F(000)	1196	
Crystal color	yellow	
Crystal size	0.311 x 0.256 x 0.167 mm^3	
Theta range for data collection	1.798 to 29.003°	
Index ranges	$-12 \le h \le 12$, $-20 \le k \le 20$, $-23 \le l \le 23$	

Reflections collected	30266
Independent reflections	6415 [R(int) = 0.0238]
Completeness to theta = 25.500°	100.0 %
Absorption correction	Numerical
Max. and min. transmission	0.9622 and 0.8095
Refinement method	Full-matrix least-squares on F^2
Data / restraints / parameters	6415 / 0 / 447
Goodness-of-fit on F^2	1.023
Final R indices [I>2sigma(I) = 6033 data]	R1 = 0.0235, wR2 = 0.0552
R indices (all data, 0.73Å)	R1 = 0.0270, wR2 = 0.0569
Largest diff. peak and hole	0.322 and -0.263 e.$Å^{-3}$

Table S2. Bond lengths [Å] and angles [°] for $[CoL^H(CH_3CN)_2][BF_4]_2$ (**1**).

Co(1)-N(5)	2.1114(18)
Co(1)-N(6)	2.1145(18)
Co(1)-N(1)	2.1342(17)
Co(1)-N(4)	2.1365(17)
Co(1)-N(3)	2.1609(17)
Co(1)-N(2)	2.1665(17)
N(1)-C(5)	1.345(3)
N(1)-C(1)	1.347(3)
N(2)-C(15)	1.478(3)
N(2)-C(6)	1.481(3)
N(2)-C(7)	1.492(3)
N(3)-C(16)	1.480(2)
N(3)-C(9)	1.483(3)
N(3)-C(8)	1.491(3)
N(4)-C(14)	1.346(3)
N(4)-C(10)	1.349(3)
N(5)-C(17)	1.132(3)
N(6)-C(19)	1.141(3)
C(1)-C(2)	1.386(3)
C(2)-C(3)	1.382(4)
C(3)-C(4)	1.382(4)
C(4)-C(5)	1.396(3)
C(5)-C(6)	1.504(3)
C(7)-C(8)	1.520(3)
C(9)-C(10)	1.505(3)
C(10)-C(11)	1.386(3)
C(11)-C(12)	1.386(3)
C(12)-C(13)	1.385(3)
C(13)-C(14)	1.386(3)
C(17)-C(18)	1.455(3)
C(19)-C(20)	1.457(3)
B(1)-F(1)	1.381(3)
B(1)-F(4)	1.384(3)
B(1)-F(2)	1.391(3)
B(1)-F(3)	1.409(3)
B(2)-F(6)	1.372(3)
B(2)-F(7)	1.378(3)
B(2)-F(5)	1.381(3)

B(2)-F(8)	1.384(3)
N(5)-Co(1)-N(6)	91.13(6)
N(5)-Co(1)-N(1)	92.59(7)
N(6)-Co(1)-N(1)	91.31(7)
N(5)-Co(1)-N(4)	89.89(7)
N(6)-Co(1)-N(4)	92.68(7)
N(1)-Co(1)-N(4)	175.26(6)
N(5)-Co(1)-N(3)	93.06(7)
N(6)-Co(1)-N(3)	169.35(7)
N(1)-Co(1)-N(3)	98.27(6)
N(4)-Co(1)-N(3)	77.55(6)
N(5)-Co(1)-N(2)	169.92(7)
N(6)-Co(1)-N(2)	93.61(7)
N(1)-Co(1)-N(2)	78.42(7)
N(4)-Co(1)-N(2)	98.76(6)
N(3)-Co(1)-N(2)	83.83(6)
C(5)-N(1)-C(1)	118.67(18)
C(5)-N(1)-Co(1)	114.24(14)
C(1)-N(1)-Co(1)	127.02(15)
C(15)-N(2)-C(6)	109.15(17)
C(15)-N(2)-C(7)	110.94(16)
C(6)-N(2)-C(7)	109.82(17)
C(15)-N(2)-Co(1)	115.05(14)
C(6)-N(2)-Co(1)	106.43(13)
C(7)-N(2)-Co(1)	105.26(12)
C(16)-N(3)-C(9)	108.91(16)
C(16)-N(3)-C(8)	110.76(15)
C(9)-N(3)-C(8)	110.07(15)
C(16)-N(3)-Co(1)	115.64(13)
C(9)-N(3)-Co(1)	106.27(12)
C(8)-N(3)-Co(1)	105.01(12)
C(14)-N(4)-C(10)	118.26(17)
C(14)-N(4)-Co(1)	127.45(14)
C(10)-N(4)-Co(1)	114.19(12)
C(17)-N(5)-Co(1)	174.57(17)
C(19)-N(6)-Co(1)	172.22(17)
N(1)-C(1)-C(2)	122.0(2)
C(3)-C(2)-C(1)	119.4(2)
C(4)-C(3)-C(2)	118.9(2)
C(3)-C(4)-C(5)	119.1(2)
N(1)-C(5)-C(4)	122.0(2)
N(1)-C(5)-C(6)	116.26(18)
C(4)-C(5)-C(6)	121.8(2)
N(2)-C(6)-C(5)	110.88(17)
N(2)-C(7)-C(8)	110.48(16)
N(3)-C(8)-C(7)	109.77(16)
N(3)-C(9)-C(10)	109.46(16)
N(4)-C(10)-C(11)	122.37(18)
N(4)-C(10)-C(9)	115.87(17)
C(11)-C(10)-C(9)	121.75(18)
C(12)-C(11)-C(10)	119.0(2)
C(13)-C(12)-C(11)	118.81(19)

C(12)-C(13)-C(14)	119.21(19)
N(4)-C(14)-C(13)	122.31(19)
N(5)-C(17)-C(18)	178.9(2)
N(6)-C(19)-C(20)	179.2(2)
F(1)-B(1)-F(4)	111.01(19)
F(1)-B(1)-F(2)	110.52(19)
F(4)-B(1)-F(2)	109.93(18)
F(1)-B(1)-F(3)	108.51(18)
F(4)-B(1)-F(3)	108.78(17)
F(2)-B(1)-F(3)	108.01(18)
F(6)-B(2)-F(7)	109.2(2)
F(6)-B(2)-F(5)	110.3(2)
F(7)-B(2)-F(5)	108.94(18)
F(6)-B(2)-F(8)	108.36(19)
F(7)-B(2)-F(8)	109.61(19)
F(5)-B(2)-F(8)	110.43(19)

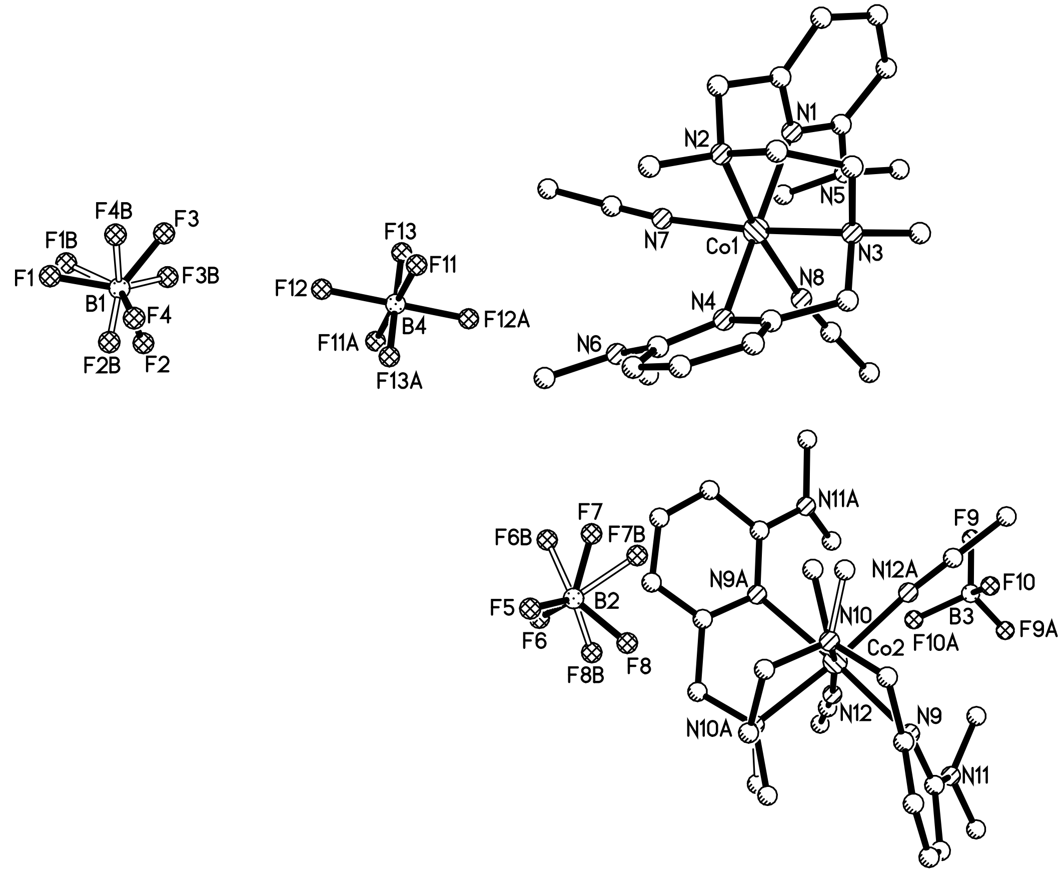

Figure S17. Ball and stick depiction of $[CoL^{DMA}(CH_3CN)_2][BF_4]_2$ (**2**) showing atomic numbering scheme.

Table S3. Crystal data and structure refinement for $[CoL^{DMA}(CH_3CN)_2][BF_4]_2$ (**2**). No metrical parameters are provided due to the poor quality of the data set; the structure is reported for connectivity only.

Empirical formula	C_{24} H_{38} B_2 Co F_8 N_8	
Formula weight	671.17	
Temperature	88(2) K	
Wavelength	0.71073 Å	
Crystal system	Monoclinic	
Space group	*C*2/*c*	
Unit cell dimensions	a = 30.4582(16) Å	α= 90°.
	b = 20.3058(11) Å	β= 120.1779(7)°.
	c = 17.0511(9) Å	γ = 90°.
Volume	9116.4(8) $Å^3$	
Z	12	
Density (calculated)	1.467 Mg/m^3	
Absorption coefficient	0.644 mm^{-1}	
F(000)	4164	
Crystal color	orange	
Crystal size	0.614 x 0.181 x 0.174 mm^3	
Theta range for data collection	1.547 to 26.396°	
Index ranges	-38 ≤ *h* ≤ 38, -25 ≤ *k* ≤ 25, -21 ≤ *l* ≤ 21	
Reflections collected	49816	
Independent reflections	9317 [R(int) = 0.0304]	
Completeness to theta = 25.500°	100.0 %	
Absorption correction	Semi-empirical from equivalents	
Max. and min. transmission	0.9450 and 0.7760	
Refinement method	Full-matrix least-squares on F^2	
Data / restraints / parameters	9317 / 0 / 513	
Goodness-of-fit on F^2	1.067	
Final R indices [I>2sigma(I) = 8006 data]	R1 = 0.1226, wR2 = 0.3233	
R indices (all data, 0.80 Å)	R1 = 0.1335, wR2 = 0.3322	
Largest diff. peak and hole	2.573 and -1.559 e.$Å^{-3}$	

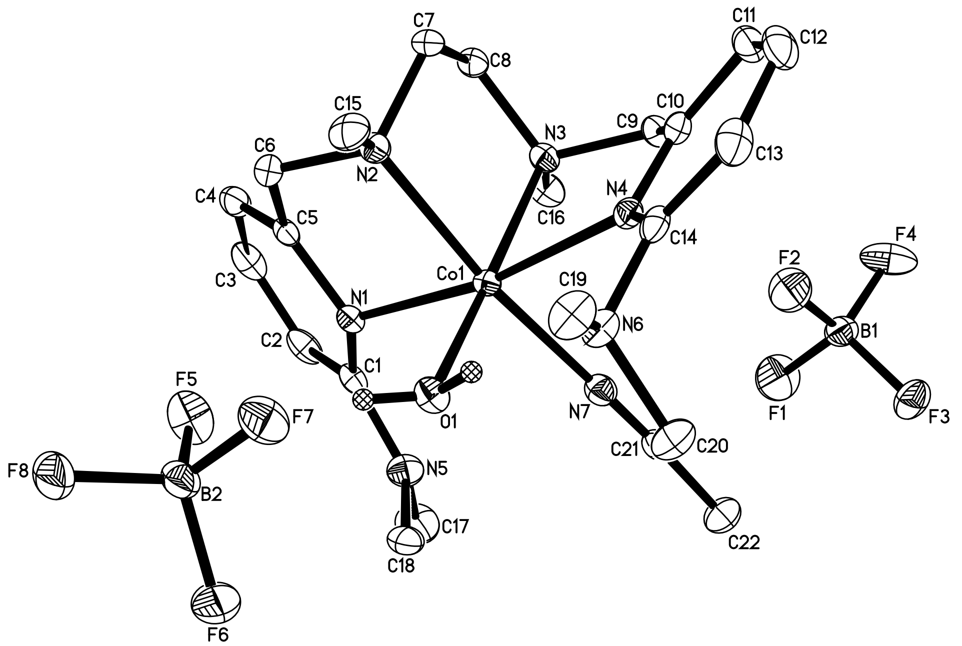

Figure S18. ORTEP of [CoL^{DMA}(CH_3CN)(H_2O)][BF_4]$_2$ showing atomic numbering scheme.

Table S4. Crystal data and structure refinement for [CoL^{DMA}(CH_3CN)(H_2O)][BF_4]$_2$.

Empirical formula	C_{22} H_{37} B_2 Co F_8 N_7 O	
Formula weight	648.13	
Temperature	88(2) K	
Wavelength	0.71073 Å	
Crystal system	Monoclinic	
Space group	*C*2/*c*	
Unit cell dimensions	a = 28.1807(18) Å	α= 90°.
	b = 13.2001(8) Å	β= 129.8712(6)°.
	c = 20.3454(13) Å	γ = 90°.
Volume	5808.5(6) Å^3	
Z	8	
Density (calculated)	1.482 Mg/m^3	
Absorption coefficient	0.672 mm^{-1}	
F(000)	2680	
Crystal color	orange	
Crystal size	0.158 x 0.134 x 0.101 mm^3	
Theta range for data collection	1.807 to 28.276°	
Index ranges	-37 ≤ *h* ≤ 37, -17 ≤ *k* ≤ 17, -26 ≤ *l* ≤ 27	
Reflections collected	34760	
Independent reflections	7125 [R(int) = 0.0366]	
Completeness to theta = 25.500°	100.0 %	
Absorption correction	Semi-empirical from equivalents	
Max. and min. transmission	0.8015 and 0.7274	
Refinement method	Full-matrix least-squares on F^2	
Data / restraints / parameters	7125 / 0 / 518	
Goodness-of-fit on F^2	1.030	
Final R indices [I>2sigma(I) = 5960 data]	R1 = 0.0288, wR2 = 0.0672	
R indices (all data, 0.75Å)	R1 = 0.0389, wR2 = 0.0716	
Largest diff. peak and hole	0.391 and -0.315 e.Å^{-3}	

Table S5. Bond lengths [Å] and angles [°] for $[CoL^{DMA}(CH_3CN)(H_2O)][BF_4]_2$.

Co(1)-O(1)	2.0571(10)
Co(1)-N(7)	2.1010(12)
Co(1)-N(3)	2.1820(11)
Co(1)-N(2)	2.1840(11)
Co(1)-N(1)	2.2057(11)
Co(1)-N(4)	2.2221(11)
N(1)-C(5)	1.3590(17)
N(1)-C(1)	1.3634(17)
N(2)-C(15)	1.4822(18)
N(2)-C(6)	1.4860(17)
N(2)-C(7)	1.4922(17)
N(3)-C(16)	1.4867(18)
N(3)-C(9)	1.4895(17)
N(3)-C(8)	1.4942(18)
N(4)-C(14)	1.3512(17)
N(4)-C(10)	1.3523(17)
N(5)-C(1)	1.3683(18)
N(5)-C(18)	1.4567(19)
N(5)-C(17)	1.4594(19)
N(6)-C(14)	1.4303(18)
N(6)-C(19)	1.4777(17)
N(6)-C(20)	1.4835(18)
N(7)-C(21)	1.1382(17)
C(1)-C(2)	1.414(2)
C(2)-C(3)	1.369(2)
C(3)-C(4)	1.393(2)
C(4)-C(5)	1.378(2)
C(5)-C(6)	1.5098(19)
C(7)-C(8)	1.513(2)
C(9)-C(10)	1.508(2)
C(10)-C(11)	1.3886(19)
C(11)-C(12)	1.383(2)
C(12)-C(13)	1.384(2)
C(13)-C(14)	1.3930(19)
C(21)-C(22)	1.4542(19)
B(1)-F(1)	1.3794(19)
B(1)-F(2)	1.3893(19)
B(1)-F(4)	1.390(2)
B(1)-F(3)	1.3917(18)
B(2)-F(8)	1.3826(18)
B(2)-F(7)	1.3873(18)
B(2)-F(6)	1.3895(18)
B(2)-F(5)	1.4170(19)
O(1)-Co(1)-N(7)	95.04(4)
O(1)-Co(1)-N(3)	171.66(4)
N(7)-Co(1)-N(3)	89.91(4)
O(1)-Co(1)-N(2)	92.41(4)
N(7)-Co(1)-N(2)	172.23(4)
N(3)-Co(1)-N(2)	82.44(4)
O(1)-Co(1)-N(1)	93.19(4)

N(7)-Co(1)-N(1)	99.24(4)
N(3)-Co(1)-N(1)	92.64(4)
N(2)-Co(1)-N(1)	82.53(4)
O(1)-Co(1)-N(4)	92.72(4)
N(7)-Co(1)-N(4)	87.53(4)
N(3)-Co(1)-N(4)	80.77(4)
N(2)-Co(1)-N(4)	89.90(4)
N(1)-Co(1)-N(4)	170.58(4)
C(5)-N(1)-C(1)	117.92(11)
C(5)-N(1)-Co(1)	106.73(8)
C(1)-N(1)-Co(1)	128.11(9)
C(15)-N(2)-C(6)	108.85(11)
C(15)-N(2)-C(7)	107.54(11)
C(6)-N(2)-C(7)	111.48(11)
C(15)-N(2)-Co(1)	117.12(9)
C(6)-N(2)-Co(1)	105.72(8)
C(7)-N(2)-Co(1)	106.15(8)
C(16)-N(3)-C(9)	108.24(10)
C(16)-N(3)-C(8)	108.06(11)
C(9)-N(3)-C(8)	111.16(10)
C(16)-N(3)-Co(1)	115.91(9)
C(9)-N(3)-Co(1)	105.55(8)
C(8)-N(3)-Co(1)	107.94(8)
C(14)-N(4)-C(10)	118.00(12)
C(14)-N(4)-Co(1)	129.56(9)
C(10)-N(4)-Co(1)	110.33(9)
C(1)-N(5)-C(18)	120.65(12)
C(1)-N(5)-C(17)	119.67(13)
C(18)-N(5)-C(17)	113.38(13)
C(14)-N(6)-C(19)	114.75(11)
C(14)-N(6)-C(20)	110.71(11)
C(19)-N(6)-C(20)	111.56(11)
C(21)-N(7)-Co(1)	176.84(11)
N(1)-C(1)-N(5)	118.22(12)
N(1)-C(1)-C(2)	120.57(13)
N(5)-C(1)-C(2)	121.21(13)
C(3)-C(2)-C(1)	119.51(14)
C(2)-C(3)-C(4)	120.06(14)
C(5)-C(4)-C(3)	117.89(14)
N(1)-C(5)-C(4)	123.61(13)
N(1)-C(5)-C(6)	115.66(12)
C(4)-C(5)-C(6)	120.63(13)
N(2)-C(6)-C(5)	114.07(11)
N(2)-C(7)-C(8)	111.27(11)
N(3)-C(8)-C(7)	111.63(11)
N(3)-C(9)-C(10)	113.28(11)
N(4)-C(10)-C(11)	122.59(13)
N(4)-C(10)-C(9)	116.26(12)
C(11)-C(10)-C(9)	121.10(13)
C(12)-C(11)-C(10)	118.74(14)
C(11)-C(12)-C(13)	119.38(14)
C(12)-C(13)-C(14)	118.89(14)
N(4)-C(14)-C(13)	122.26(13)

N(4)-C(14)-N(6)	115.25(11)
C(13)-C(14)-N(6)	122.48(13)
N(7)-C(21)-C(22)	177.32(15)
F(1)-B(1)-F(2)	110.28(14)
F(1)-B(1)-F(4)	109.59(13)
F(2)-B(1)-F(4)	109.84(13)
F(1)-B(1)-F(3)	109.06(12)
F(2)-B(1)-F(3)	109.08(13)
F(4)-B(1)-F(3)	108.97(13)
F(8)-B(2)-F(7)	111.03(12)
F(8)-B(2)-F(6)	109.75(12)
F(7)-B(2)-F(6)	110.02(13)
F(8)-B(2)-F(5)	109.22(13)
F(7)-B(2)-F(5)	107.98(12)
F(6)-B(2)-F(5)	108.78(12)

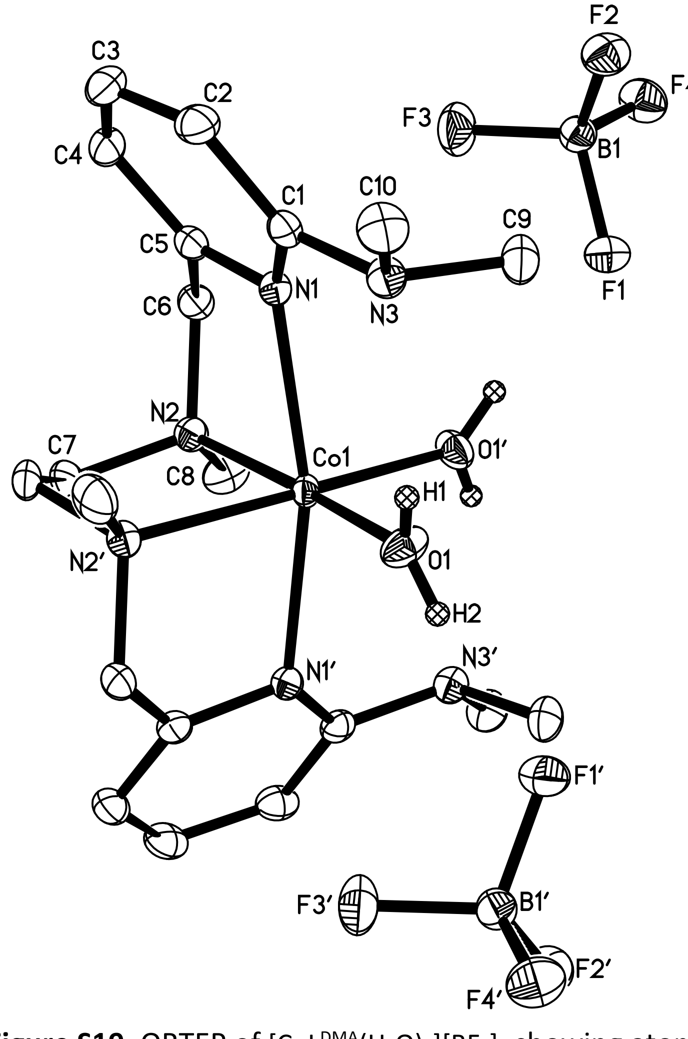

Figure S19. ORTEP of $[CoL^{DMA}(H_2O)_2][BF_4]_2$ showing atomic numbering scheme.

Table S6. Crystal data and structure refinement for $[CoL^{DMA}(H_2O)_2][BF_4]_2$.

Empirical formula	$C_{20} H_{36} B_2 Co F_8 N_6 O_2$	
Formula weight	625.10	
Temperature	88(2) K	
Wavelength	0.71073 Å	
Crystal system	Monoclinic	
Space group	*P*2/*n*	
Unit cell dimensions	a = 8.3869(7) Å	α= 90°.
	b = 7.9947(6) Å	β= 90.1405(11)°.
	c = 19.4819(16) Å	γ = 90°.
Volume	1306.27(18) Å^3	
Z	2	
Density (calculated)	1.589 Mg/m^3	
Absorption coefficient	0.746 mm^{-1}	
F(000)	646	
Crystal color	pink	
Crystal size	0.336 x 0.255 x 0.200 mm^3	
Theta range for data collection	2.091 to 28.990°	
Index ranges	$-11 \le h \le 11$, $-10 \le k \le 10$, $-26 \le l \le 25$	
Reflections collected	15619	
Independent reflections	3301 [R(int) = 0.0249]	
Completeness to theta = 25.500°	100.0 %	
Absorption correction	Semi-empirical from equivalents	
Max. and min. transmission	0.7458 and 0.6750	
Refinement method	Full-matrix least-squares on F^2	
Data / restraints / parameters	3301 / 0 / 249	
Goodness-of-fit on F^2	1.031	
Final R indices [I>2sigma(I) = 2937 data]	R1 = 0.0270, wR2 = 0.0666	
R indices (all data, 0.73Å)	R1 = 0.0320, wR2 = 0.0693	
Largest diff. peak and hole	0.385 and -0.222 e.Å^{-3}	

Table S7. Bond lengths [Å] and angles [°] for $[CoL^{DMA}(H_2O)_2][BF_4]_2$.

Co(1)-O(1)#1	2.0352(10)
Co(1)-O(1)	2.0352(10)
Co(1)-N(2)#1	2.1847(11)
Co(1)-N(2)	2.1847(11)
Co(1)-N(1)#1	2.2426(11)
Co(1)-N(1)	2.2426(11)
N(1)-C(5)	1.3530(17)
N(1)-C(1)	1.3535(17)
N(2)-C(6)	1.4833(17)
N(2)-C(8)	1.4857(18)
N(2)-C(7)	1.4912(17)
N(3)-C(1)	1.4074(17)
N(3)-C(10)	1.4682(17)
N(3)-C(9)	1.4718(17)
C(1)-C(2)	1.4051(18)
C(2)-C(3)	1.380(2)
C(3)-C(4)	1.387(2)
C(4)-C(5)	1.3853(19)

C(5)-C(6)	1.5040(19)
C(7)-C(7)#1	1.520(3)
B(1)-F(2)	1.3892(17)
B(1)-F(3)	1.3909(17)
B(1)-F(4)	1.3920(17)
B(1)-F(1)	1.4107(17)

O(1)#1-Co(1)-O(1)	96.38(6)
O(1)#1-Co(1)-N(2)#1	173.08(4)
O(1)-Co(1)-N(2)#1	90.16(4)
O(1)#1-Co(1)-N(2)	90.16(4)
O(1)-Co(1)-N(2)	173.08(4)
N(2)#1-Co(1)-N(2)	83.40(6)
O(1)#1-Co(1)-N(1)#1	96.05(4)
O(1)-Co(1)-N(1)#1	93.23(4)
N(2)#1-Co(1)-N(1)#1	81.28(4)
N(2)-Co(1)-N(1)#1	88.30(4)
O(1)#1-Co(1)-N(1)	93.23(4)
O(1)-Co(1)-N(1)	96.05(4)
N(2)#1-Co(1)-N(1)	88.30(4)
N(2)-Co(1)-N(1)	81.28(4)
N(1)#1-Co(1)-N(1)	166.06(5)
C(5)-N(1)-C(1)	118.03(11)
C(5)-N(1)-Co(1)	107.65(8)
C(1)-N(1)-Co(1)	127.79(8)
C(6)-N(2)-C(8)	107.40(11)
C(6)-N(2)-C(7)	111.94(10)
C(8)-N(2)-C(7)	107.73(11)
C(6)-N(2)-Co(1)	104.72(8)
C(8)-N(2)-Co(1)	118.16(8)
C(7)-N(2)-Co(1)	106.97(8)
C(1)-N(3)-C(10)	115.43(11)
C(1)-N(3)-C(9)	113.56(11)
C(10)-N(3)-C(9)	110.68(11)
N(1)-C(1)-C(2)	121.21(12)
N(1)-C(1)-N(3)	116.16(11)
C(2)-C(1)-N(3)	122.62(12)
C(3)-C(2)-C(1)	119.56(13)
C(2)-C(3)-C(4)	119.38(13)
C(5)-C(4)-C(3)	118.20(13)
N(1)-C(5)-C(4)	123.42(13)
N(1)-C(5)-C(6)	115.58(11)
C(4)-C(5)-C(6)	121.00(12)
N(2)-C(6)-C(5)	112.67(11)
N(2)-C(7)-C(7)#1	112.87(9)
F(2)-B(1)-F(3)	110.13(12)
F(2)-B(1)-F(4)	109.40(11)
F(3)-B(1)-F(4)	109.80(12)
F(2)-B(1)-F(1)	108.89(11)
F(3)-B(1)-F(1)	108.87(12)
F(4)-B(1)-F(1)	109.72(11)

Symmetry transformations used to generate equivalent atoms: #1 -x+1/2,y,-z+1/2

References

1. D. Coucouvanis, in *Inorg. Synth.*, John Wiley & Sons, Inc., 2002, DOI: 10.1002/0471224502.ch2, pp. 75-121.
2. J. F. Khosrowabadi Kotyk, J. W. Ziller and J. Y. Yang, *J. Coord. Chem.*, 2016, **69**, 1990-2002.
3. C. Tsay and J. Y. Yang, *J. Am. Chem. Soc.*, 2016, DOI: 10.1021/jacs.6b05851.
4. A. B. P. Lever, *Inorganic Electronic Spectroscopy*, Elsevier, 1984.
5. L. C. Anderson and N. V. Seeger, *J. Am. Chem. Soc.*, 1949, **71**, 340-342.
6. L. Que Jr, *Physical Methods in Bioinorganic Chemistry*, University Science Books, 2000.
